# 2-methoxyestradiol inhibits the malignant behavior of triple negative breast cancer cells by altering their miRNome

**DOI:** 10.3389/fonc.2024.1371792

**Published:** 2024-09-12

**Authors:** Ramadevi Subramani, Animesh Chatterjee, Diego A. Pedroza, Seeta Poudel, Preetha Rajkumar, Jeffrey Annabi, Elizabeth Penner, Rajkumar Lakshmanaswamy

**Affiliations:** ^1^ Center of Emphasis in Cancer Research, Department of Molecular and Translational Medicine, Texas Tech University Health Sciences Center El Paso, Paul L. Foster School of Medicine, El Paso, TX, United States; ^2^ Francis Graduate School of Biomedical Sciences, Texas Tech University Health Sciences Center, El Paso, TX, United States; ^3^ College of Osteopathic Medicine, Rocky Vista University, Ivins, UT, United States

**Keywords:** 2-methoxyestradiol, microRNA, cell cycle, invasion, migration, proliferation, apoptosis, triple negative breast cancer

## Abstract

**Background:**

Triple-negative breast cancer (TNBC) is a subtype of breast cancer with no effective targeted treatment currently available. Estrogen and its metabolites influence the growth of mammary cancer. Previously, we demonstrated the anti-cancer effects of 2-methoxyestradiol (2ME2) on mammary carcinogenesis.

**Materials and methods:**

In the present study, we investigated the effects of 2ME2 on TNBC cells. TNBC (MDA-MB-231 and MDA-MB-468) and non-tumorigenic breast (MCF10A) cell lines were used to determine the effects of 2ME2 on cell proliferation (3-(4,5-dimethylthiazol-2-yl)-5-(3-carboxymethoxyphenyl)-2-(4-sulfophenyl)-2H-tetrazolium; MTS assay), cell cycle (flow cytometric assay), migration (transwell migration assay), invasion (matrigel invasion assay), apoptosis (annexin V/propidium iodide assay), colony formation (soft agar assay), and miRNome (human miRNA profiling array). The miRNome data were analyzed using the c-BioPortal and Xena platforms. Moreover, Kyoto Encyclopedia of Genes and Genomes, Gene Ontology, and reactome pathway analyses were performed.

**Results:**

We found that 2ME2 effectively inhibited cell proliferation and induced apoptosis. Furthermore, 2ME2 treatment arrested TNBC cells in the S-phase of the cell cycle. Treatment with 2ME2 also significantly decreased the aggressiveness of TNBC cells by inhibiting their migration and invasion. In addition, 2ME2 altered the miRNA expression in these cells. In silico analysis of the miRNome profile of 2ME2-treated MDA-MB-468 cells revealed that miRNAs altered the target genes involved in many different cancer hallmarks.

**Conclusion:**

2ME2 inhibits triple negative breast cancer by impacting major cellular processes like proliferation, apoptosis, metastasis, etc. It further modifies gene expression by altering the miRNome of triple negative breast cancer cells. Overall, our findings suggest 2ME2 as a potent anti-cancer drug for the treatment of TNBC.

## 1 Introduction

Triple-negative breast cancer (TNBC) is an aggressive and highly metastatic disease with a poor prognosis. This lethal breast cancer subtype is histologically classified as estrogen receptor (ER), progesterone receptor (PR), and human epidermal growth factor receptor 2 [HER2]-negative (1, 2) and accounts for approximately 20% of all diagnosed breast cancer cases (3). TNBC has worse prognosis than other breast cancer subtypes (3). It is commonly diagnosed in younger women (4) and poses a high risk of recurrence (4). Metastasis is the main cause of breast-cancer-associated mortality. Treatment options are limited because of the lack of specific targets for TNBC. The two major subtypes of TNBC cell lines are basal A and basal B (5). These cell line subtypes have clear distinguishing features between them in the expression of cytokeratins, integrins, claudins, mesenchymal and stem cell markers (6–9). Currently, chemotherapy is the only approved systemic treatment for TNBC that shows limited benefits in terms of overall survival and toxicity (10, 11). Extensive efforts have been made to develop effective drugs for the treatment of high-risk TNBC. However, non-toxic and effective therapies are urgently required to treat advanced-stage TNBC.

2-Methoxyestradiol (2ME2) is a primary endogenous metabolite of 17β-estradiol (12). Its affinity for ERs is weak compared to that of its parent compound and 17β-estradiol and its other derivatives (12). Its effect is independent of the estrogen responsiveness of the cells (13). Several studies have demonstrated the impact of 2ME2 on hypoxia, angiogenesis, vascularization, and drug resistance (14). The compound 2ME2 has demonstrated anti-cancer effects across various cancer types, including breast cancer (15–17), ovarian cancer (18), prostate cancer (19), osteosarcoma (20), leukemia (21), cervical adenocarcinoma (22), melanoma (23), and sarcoma (24). These effects are attributed to its capacity to induce apoptosis, cause cell cycle arrest, instigate nitro oxidative damage, promote autophagy, and inhibit invasion, angiogenesis, and overall tumor growth. Notably, oral administration of 2ME2 does not cause any significant side effects in xenograft and metastatic disease models (25, 26). Over the last decade, 2ME2 has gained attention for its notable anti-cancer activity and potential cardiovascular benefits (27). Phase I and II clinical trials have been conducted to evaluate the antiproliferative and antiangiogenic effects of 2ME2 on multiple myeloma, glioblastoma multiforme, and carcinoid, prostate, and breast tumors (13, 28).

2ME2 has been shown to inhibit the proliferation of cancer cells by inducing apoptosis (15, 29), through upregulation of death receptor 5 (30) targeting cells in the G2/M phase of cell cycle (31, 32), inhibiting microtubule assembly (25) and also inhibit angiogenesis (33). However, the exact molecular mechanisms by which 2ME2 exerts its pleiotropic effects on breast cancer cells remain unclear. Therefore, in this study, we aimed to elucidate the signaling pathways regulated by miRNAs that are associated with the anti-cancer effects of 2ME2 on TNBC cells. MicroRNAs (miRNAs) are a class of small non-coding RNAs that regulate the expression of several genes in humans, plants, and microbes via RNA silencing and post-translational regulation (34). In cancer, miRNAs function as either tumor suppressors or oncogenes, ultimately affecting their target gene expression (35). Differential expression of miRNAs regulates different pathways such as apoptosis, cancer-related, and cell proliferation pathways. It has been demonstrated that TNBC subtypes A and B cells had a distinct microRNA profiles resulting in differential metastatic potentials (5, 6). In this study, we observed a change in the global human miRNome profile of 2ME2-treated TNBC cells. Furthermore, the altered miRNome influenced various cancer pathways, including the proliferation, metastasis, cell cycle, apoptosis, mammalian target of rapamycin (mTOR), focal adhesion, p53, carbohydrate digestion and absorption, insulin, cell cycle, Janus kinase (JAK)-signal transducer and activator of transcription (STAT), ErbB, adherens, neurotrophin, T cell receptor, and endocytosis pathways. Our findings provide insights into the effects of 2ME2 on TNBC, suggesting its potential as a drug for the treatment of this aggressive type of breast cancer.

## 2 Materials and methods

### 2.1 Cell lines

The non-malignant breast epithelial cell line, MCF-10A (Cat# CRL-10317), and triple-negative metastatic breast cancer cell lines MDA-MB-231 (Cat # HTB-26) and MDA-MB-468 (Cat # HTB-132) were purchased from the American Type Culture Collection (ATCC, Manassas, VA, USA). MCF-10A cells were grown in a mammary epithelial cell basal medium supplemented with 5% horse serum, 0.5 mL epidermal growth factor, 0.5 mL insulin, 0.5 mL hydrocortisone, 2 mL bovine pituitary extract (all from Lonza, Clonetics, MEGM SingleQuots kit), 10 U/mL penicillin, and 10 mg/mL streptomycin (Gibco, Life Technologies). MDA-MB-231 and MDA-MB-468 cells were maintained in the Roswell Park Memorial Institute (RPMI)-1640 medium (Invitrogen Corporation, Carlsbad, CA, USA) supplemented with 10% heat-inactivated fetal bovine serum (FBS), 10 U/mL penicillin, and 10 mg/mL streptomycin (Gibco, Life Technologies). Phenol red-free RPMI 1640 medium supplemented with 10% charcoal-stripped serum (Equitech-Bio, Kerrville, TX, USA) was utilized for all cell lines in this study. This medium was applied 48 hours before 2ME2 treatment to prevent estrogen interference and ensure the accuracy of the observed results. Mycoplasma contamination was tested using the MycoSEQ Plus Mycoplasma Detection Kits (ThermoFisher Scientific Cat # A55124). All cells were maintained at 37°C in a 5% CO_2_ atmosphere.

### 2.2 Cell proliferation assay

An *in vitro* cell viability assay was performed as previously described (36). Briefly, MDA-MB-231, MDA-MB-468, and MCF-10A cells were seeded in a 96-well plate at a density of 5 × 10^3^ cells/well and incubated at 37°C. After 24 h, the cells were treated with different concentrations of 2ME2 (0, 1, 2, 5, 7.5, 10, and 20 μM) and incubated for different time periods from 24 to 72 h at 37°C. After incubation, twenty microliters of the Cell Titer 96 Aqueous One Solution Reagent (Promega) was added to each well of the 96-well assay plate containing the samples in 100 μL of the culture medium. The plates were incubated at 37°C for 4 h in a humidified 5% CO_2_ atmosphere. Cell viability was determined by measuring the absorbance at 490 nm using a 96-well microplate reader (CLARIOstar; BMG LABTECH, Cary, NC, USA).

### 2.3 Immunoblotting

Western blotting was performed to quantify the expression of various proteins. Mammalian protein extraction reagent (Cat. no. 78501; Thermo Scientific, Rockford, IL, USA) was used to extract the total protein from whole cell lysates. Proteins were separated using SDS-PAGE and then transferred to polyvinylidene fluoride (PVDF; cat. no. IPVH00010; EMD Millipore) membranes. The membranes were blocked for 1 h with 5% bovine serum albumin (Cat. no. A7906; Sigma-Aldrich, St Louis, MO, USA) prepared in Tris-buffered saline with 0.1% Tween-20. Then, the blots were incubated with primary antibodies (pAKT, AKT, pmTOR, mTOR, pP70S6K, P70S6K, E-cadherin, N-cadherin, vimentin, slug, zinc finger E-box-binding homeobox 1 [Zeb1], cyclin-dependent kinase [CDK]4, CDK6, cyclin D1, p21, cleaved poly[ADP ribose] polymerase [PARP], cleaved caspase 3, Bcl-associated killer (BAK), Bcl-2, and β-actin) overnight at 4°C. Details regarding antibodies and their dilutions are provided in the **Supplementary Materials** as **Table 1**. After washing thrice with TBST, the blots were incubated with the appropriate horseradish peroxidase-conjugated secondary antibodies to detect the primary antibodies and visualized using enhanced chemiluminescence.

**Table 1:** List of antibodies and dilutions.

Antibody	Company	Dilution	Molecular weight (kDa)
pAKT S473	Cell Signaling 4060	1:1000	60
AKT1	Santa cruz 5298	1;500	62
pmTOR S2448	Cell Signaling 5536	1:500	289
mTOR S2448	Cell Signaling 4517	1:500	289
pP70S6K	Cell Signaling 9206	1:500	70-85
P70S6K	Cell Signaling 9202	1:500	70-85
E-cadherin	Cell Signaling 3195	1:500	135
N-cadherin	Cell Signaling 13116	1:500	140
Vimentin	Cell Signaling 5741	1:500	57
Slug	Cell Signaling 9585	1:500	30
Zeb1	Cell Signaling 3396	1:500	200
CDK4	BD Biosciences 610147	1:1000	33
CDK6	Cell Signaling 30483	1:500	36
Cyclin D1	Santa cruz 717	1:1000	37
P21	Abcam 7960	1:100	21
Cleaved PARP/PARP (Asp 214)	Cell Signaling 5625	1:1000	89-116
BAK	Cell Signaling 3814	1:1000	25
Bcl2	Santa cruz 783	1:500	26
Cleaved Caspase 3	Cell Signaling 9661	1:500	17-19
β-Actin	Sigma A1978	1:1000	42

Image Studio software was used to quantify protein expression from digitally imaged Western blots. An equal dimension rectangle tool from the analysis ribbon was used to draw lanes around the entire region where bands were present, allowing for measurement of all protein signals. Background subtraction was applied to each band within a lane to eliminate background noise from the calculations. The intensity values of the bands corresponding to the protein of interest were normalized to their respective β-actin intensity values. The data are represented as the percentage of protein expression normalized to β-actin.

### 2.4 Cell cycle analysis

MDA-MB-468 cells in logarithmic growth phase were harvested and seeded in a 6-well plate at a density of 5.0 × 10^5^ cells/well. After overnight culture, the cells were treated with 5 μM 2ME2 for 24 h, harvested, and stained with propidium iodide. The percentage of cells in each phase of the cell cycle was analyzed via flow cytometry using FACS Accuri C6 flow cytometer (BD Biosciences San Jose, CA, USA).

### 2.5 Apoptosis analysis

MDA-MB-468 cells were seeded in a 6-well plate at a density of 2.0 × 10^5^ cells/well and treated with 5 μM 2ME2 for 24, 48, and 72 h. Apoptosis was detected using the Annexin V-FITC Apoptosis Detection Kit I and analyzed using the FACS Accuri C6 flow cytometer (BD Biosciences San Jose, CA, USA), according to the manufacturers’ instructions.

### 2.6 Migration assay

The migration assay was performed using 2ME2 (2 and 5 μM)-treated MDA-MB-468 cells. Cells (5 × 10^3^ cells/well) were seeded in a serum free medium in the top chamber of the transwell with a non-coated polycarbonate membrane (6.5 mm diameter insert, 8.0 μm pore size; Corning Incorporated). In the lower chamber, RPMI-1640 medium with 10% FBS was added. The cells were incubated for 48 h at 37°C and 5% CO_2_. Migrating cells on the lower surface of the membrane were fixed with 5% formalin and stained with 0.2% crystal violet. Non-migrating cells on the upper side of the insert were wiped off using a cotton swab. Images of the migrated cells were captured and counted in a blinded manner in five different microscopic fields at 20× magnification.

### 2.7 Invasion assay

Invasive ability of MDA-MB-468 breast cancer cells was further assessed after treatment with 2ME2 (5 μM) using a matrigel-based invasion assay. Cells were seeded in a serum-free medium at a density of 0.5 × 10^5^ cells/well in the upper chamber of a 6.5-mm transwell with 8.0 mm pore polycarbonate membrane inserts (Corning) coated with matrigel (Cat. no. 354234; BD Biosciences). The lower chamber was filled with the medium containing 10% FBS and incubated for 48 h at 37°C. After incubation, matrigel from the upper chamber was gently scraped using Q-tips. The cells invaded on the other side of the membrane facing the lower chamber were rinsed with 1× PBS and the cells were fixed with 4% paraformaldehyde. The invaded cells were then stained with 0.1% crystal violet for 2 h. Then, the insert wells were washed with tap water and imaged under an inverted phase contrast microscope (Eclipse Ti; Nikon).

### 2.8 Colony formation assay

Colony formation assay was performed to assess the clonogenic ability of single cells under anchorage-independent growth conditions. MDA-MB-468 cells (2 × 10^4^) were seeded in 60-mm dishes containing a top layer of 0.7% agarose and bottom layer of 1% agar. The cells were treated with 2 and 5 μM 2ME2 for 24 h and incubated at 37°C for four weeks, and stained with 0.2% crystal violet. Stained colonies were imaged using an inverted microscope. Colonies containing more than 50 cells were manually counted.

### 2.9 Human miRNome profiling

miRNA profiling was performed using the Qiagen Human miRNome miScript miRNA PCR Array (Cat. no. MIHS-216Z). First, RNA was extracted from MDA-MB-468 cells using TRIzol (Life Technologies) after treatment with 2ME2 (5 μM) for 24 h. Then, whole human miRNome profiling PCR array was performed using the miScript SYBR Green PCR Kit (Qiagen). miRNA expression data were assessed using miRNet (http://www.mirnet.ca/).

### 2.10 RTqPCR analysis

Total RNA was isolated from MDA-MB-468 and MDA-MB-231 cells after treatment with 2ME2 (5 μM) for 24 h using the Trizol reagent (Life Technologies). The top 5 up-regulated and down-regulated miRNAs and its target genes were validated using RTqPCR. The appropriate primers used for each miRNA and its target genes is provided in the **Supplementary Materials** section as **Tables 2** and **3**. The analyses were performed based on the comparative Ct method (2−ΔΔCt).

**Table 2:** List of miRNA primers.

Top 5 up regulated miRNAs by 2ME2	Forward	Reverse
hsa-miR-4326	TGTTCCTCTGTCTCCCAG	GAACATGTCTGCGTATCTC
hsa-miR-646	5'-ACACTCCAGCTGGGAAGCAGCTGCCTC-3'	5'-
hsa-miR-600	CCACCTCTACGCATCATTCA	CCAAGCTCGTCTGGTTCTC
hsa-miR-2117	TGTTCTCTTTGCCAAGGAC	GAACATGTCTGCGTATCTC
hsa-miR-3200-5p	AATCTGAGAAGGCGCACAAG	GAACATGTCTGCGTATCTC
Top 5 downregulated miRNAs by 2ME2	Forward	Reverse
hsa-miR-19a-3p	GCGTGTGCAAATCTATGCAA	AGTGCAGGGTCCGAGGTATT
hsa-miR-15a-5p	5′-TAGCAGCACATAATGGTTTGTG-3′	5′-GAACATGTCTGCGTATCTCAC-3′
hsa-miR-21-3p	GACCCAACACCAGTCGATG	TCCTCCTCTCCTTCCTTCTC
hsa-miR-19b-3p	GTGCAAATCCATGCAAAACTGA	GTGCAGGGTCCGAGGTGCT
hsa-miR-96-5p	ATGCTTTCTCAACTTGTTGG	TCACCG CTCTTGGCCGTCACA

**Table 3:** List of miRNA target genes primers.

Genes	Forward	Reverse
PIK3R1	TGGACGGCGAAGTAAAGCATT	AGTGTGACATTGAGGGAGTCG
HSP90AA1	CAGAGGCGGACAAGAACGACAAG	GATCCTGTTGGCGTGCGTCTG
ERBB2	GGAAGTACACGATGCGGAGACT	ACCTTCCTCAGCTCCGTCTCTT
CCND1	TCTACACCGACAACTCCATCCG	TCTGGCATTTTGGAGAGGAAGTG
PTPRF	ATGTCATCGCCTACGACCACTC	GTGGCGATGTAGGCATTCTGCT
CDC42	TGACAGATTACGACCGCTGAGTT	GGAGTCTTTGGACAGTGGTGAG
HSP90B1	GGAGAGTCGTGAAGCAGTTGAG	CCACCAAAGCACACGGAGATTC
HSPA8	ACTCCAAGCTATGTCGCCTTT	TGGCATCAAAAACTGTGTTGGT
PRKAR1A	GCAGGCGAGCTATTAGTTTA	CATCCATCTCCTATCCCCTTT
AKT1	TGGACTACCTGCACTCGGAGAA	GTGCCGCAAAAGGTCTTCATGG
RAB5B	GGAGACTTCAGCCAAGACAGCT	ACACTGGCTCTTGTTCTGCTGG
FASN	TTCTACGGCTCCACGCTCTTCC	GAAGAGTCTTCGTCAGCCAGGA
GRB2	GAAATGCTTAGCAAACAGCGGCA	TCCATCTCGGAGCACCTTGAAG
GAPDH	GATGCTGGCGCTGAGTACG	GCTAAGCAGTTGGTGGTGC

### 2.11 Kyoto encyclopedia of genes and genomes, gene ontology and reactome pathway analyses

KEGG pathway analysis comprises pathway maps that help our understanding of molecular interactions, reactions, and relational networks in various categories, including Metabolism, Genetic Information Processing, Environmental Information Processing, Cellular Processes, Organismal Systems, Human Diseases, Drug Development, etc. Altered pathways were studied using KEGG and GO after 2ME2 treatment. Enriched pathway analysis was performed via hierarchical genome-wide visualization using the Reactome Analysis Tool (http://reactome.org). Significantly enriched pathways are shown in yellow, whereas less significantly enriched pathways are shown in gray. Then, miRNA target genes were uploaded to the Reactome database and significantly enriched pathways had a false discovery rate < 0.05.

### 2.12 In silico analysis of 2ME2-induced genetic alterations

Using the miRNA target genes, we explored the possible genetic alterations, including truncation, in-frame mutations, missense mutations, gene amplification (overexpression), and deletions, caused by 2ME2 treatment. Our differentially expressed gene dataset was uploaded to the cBioPortal (http://cbioportal.org/) database. In the cBioPortal database, we validated genes using The Cancer Genome Atlas (TCGA) and analyzed the overall survival of patients with either high or low expression of miRNA target genes after 2ME2 treatment. Kaplan–Meier plots were generated using breast carcinoma samples from TCGA database using Kmplot.com and UALCAN. Genetic alterations were visualized using Oncoprint diagrams of breast carcinoma samples. Using the Xena platform (http://xenabrowser.net), we further determined the changes in the gene expression profiles of TCGA breast cancer samples after 2ME2 treatment.

### 2.13 Statistical analyses

All data were analyzed using GraphPad Prism 9 (Dotmatics). All ***in vitro*** experiments were repeated at least three times and had 3–6 replicates. Student’s *t*-test was performed to compare between two groups and one-way ANOVA and Tukey ***post hoc*** test were used for analysis when more than two groups were compared, and statistical significance was set at *P* < 0.05.

## 3 Results

### 3.1 2ME2 inhibits TNBC cell growth and proliferation

**Figure 1:**
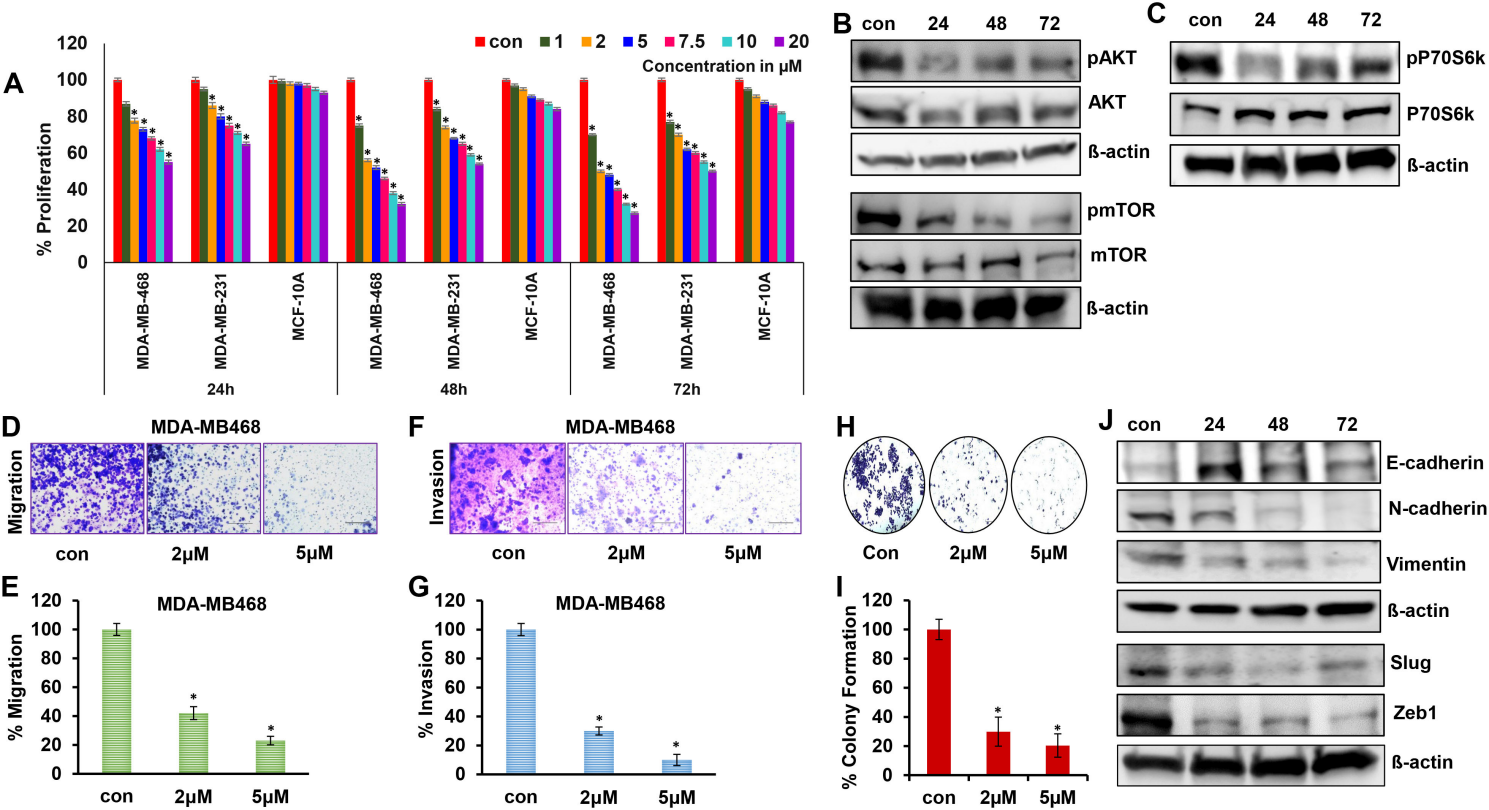
2ME2 inhibits breast cancer cell proliferation and metastasis. **(A)** Cell viability of MDA-MB-468, MDA-MB-231 and MCF-10A cells treated with 1-20μM of 2ME2 for 24, 48 and 72h. **(B, C)** Expression level of proliferative markers in MDA-MB-468 cells at different time points of treatment with 5μM of 2ME2. **(D, E)** Effect of 2ME2 on migratory capacity of MDA-MB-468 cells. **(F, G)** Effect of 2ME2 on invasive ability of MDA-MB-468 cells. **(H, I)** Colony formation ability of MDA-MB-468 cells with 2ME2 treatment. **(J)** Expression level of EMT markers in 2ME2 treated MDA-MB-468 cells at different time points. *P < 0.05.

To determine the efficacy of 2ME2 for TNBC treatment, we evaluated its effects on the growth and proliferation of TNBC cells. TNBC (MDA-MB-231 and MDA-MB-468) and non-malignant breast epithelial (MCF-10A) cell lines were treated with various concentrations of 2ME2 (0, 1, 2, 5, 7.5, 10, and 20 μM). High doses of 2ME2 increased the death of TNBC cells but did not exert any significant effects on MCF10A cells. After 24-h treatment, none of the tested doses induced 50% cell death in the cell lines. Treatment with 2 and 5 μM of 2ME2 for 48 h inhibited the proliferation of MDA-MB-468 cells by approximately 50% (**Figure 1A**). Even though 2 μM of 2ME2 was effective 5 μM dose was closest to the IC_50_ value, so we chose to use this dose for our experiments. In contrast, even high doses of 2ME2 (20 μM) did not have a major impact on MCF-10a cell proliferation. MDA-MB-231 cell proliferation was inhibited by 2ME2 in a dose-dependent manner but higher doses of 2ME2 were required (**Figure 1A**). Therefore, MDA-MB-468 cells were used for subsequent analyses. AKT/mTOR signaling pathway is hyperactive in cancer cells, facilitating cancer cell growth and proliferation (37, 38). Here, treatment with 5 μM 2ME2 for 24 h decreased the phosphorylation of AKT and mTOR (**Figure 1B** and **Supplementary Figure 1A**). We also observed decreased phosphorylation of AKT in MDA-MB-231 cells treated with 2ME2 (**Supplementary Figures 1B, C**). Next, we focused on the ribosomal protein kinase, P70S6K, which acts downstream of mTOR and regulates cell growth via G1 phase cell cycle progression (37, 39). Here, the active form of P70S6K was minimally expressed in 2ME2-treated cells (**Figure 1C** and **Supplementary Figure 1D**). 2ME2 effectively inhibited cell proliferation by reducing the activation of AKT, mTOR and P70s6Kinase. However, further analysis is needed to better understand the involvement of the PI3K/AKT signaling pathway by 2ME2 as a crucial mechanism in controlling TNBC proliferation.

### 3.2 2ME2 inhibits the metastasis of TNBC cells

Metastatic characteristics of cancer cells are defined by their ability to migrate, invade, and form a niche (colony) in other tissues/organs (40). This makes cancer more aggressive and difficult to treat. In this study, the metastatic characteristics of TNBC cells were assessed in the presence of 2ME2. We found that TNBC cell migration was inhibited by 58 and 77% after treatment with 2 and 5 μM 2ME2, respectively (**Figures 1D, E**). Invasion and colony formation were reduced in 2ME2-treated MDA-MB-468 cells than in the untreated cells (**Figures 1F–I**). Similarly, MDA-MB-231 cells treated with 2ME2 showed a significant reduction in migration and invasion capabilities (**Supplementary Figures 1E, F**) respectively. Change in cellular morphology from epithelial to mesenchymal, known as the epithelial–mesenchymal transition, is a prerequisite for metastasis (41). Treatment with 2ME2 decreased the levels of mesenchymal markers, such as N-cadherin, vimentin, slug, and Zeb1, but increased the levels of the epithelial marker, E-cadherin (**Figure 1J** and **Supplementary Figure 1G**). MDA-MB-231 cells also responded similarly to 2ME2 in terms of E-cadherin and N-cadherin regulation (**Supplementary Figures 1H–J**) respectively. These data demonstrate the potent roles of 2ME2 in inhibiting the aggressiveness and metastatic characteristics of TNBC cells.

### 3.3 2ME2 inhibits the cell cycle and induces apoptosis

**Figure 2:**
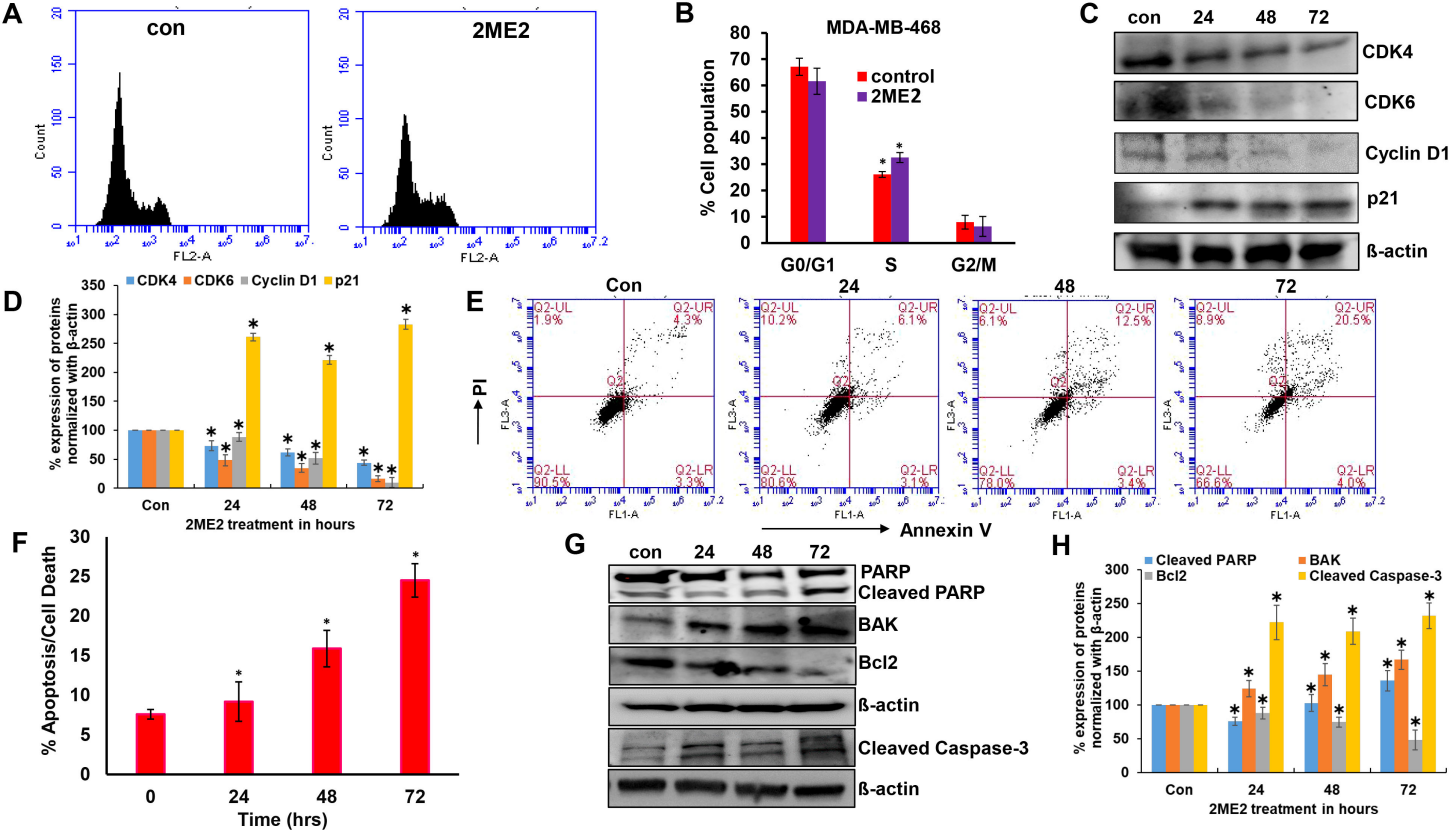
2ME2 induces cell cycle arrest and Apoptosis. **(A, B)** Cell cycle analysis of 2ME2 treated MDA-MB-468 cells. **(C, D)** Cell cycle regulators were assessed in 2ME2 treated MDA-MB-468 cells at 24, 48 and 72h using Western blot and densitometric analysis. **(E)** Apoptosis analysis of 2ME2 treated MDA-MB-468 cells at various time points, **(F)** Graphical representation of percentage apoptosis/cell death in 2ME2 treated MDA-MB-468 cells. **(G, H)** Expression levels and densitometric analysis of pro and anti-apoptotic markers in 2ME2 treated MDA-MB-468 cells at different time points. *P < 0.05.

Ribosomal protein P70S6K is active during the G1 phase of cell cycle and regulates the cellular machinery via protein synthesis required for cell division (42). P70S6K phosphorylates and regulates the functions of the 40s ribosomal protein (S6), eukaryotic initiation factor 4B, and eukaryotic elongation factor 2 kinase that are required for protein synthesis (43–45). The potent role of P70S6K in the cell cycle and its low activation in 2ME2-treated cells lead us to further analyze the cell cycle in 2ME2-treated MDA-MB-468 cell lines. We analyzed the effect of 2ME2 on the cell cycle using flow cytometry. Studies on many cancer types, including breast cancer, have reported that 2ME2 accumulates and arrests cells in almost all phases of the cell cycle, including the G0/G1, G2/M, M phases (46–49). However, its exact roles during specific phases of the cell cycle remain unknown. Our analysis of the cell cycle in 2ME2-treated MDA-MB-468 cells revealed minute changes in the cell population in S-phase (6.46% more cell population was arrested in the S-phase in 2ME2-treated cells compared to that in the non-treated cells) (**Figures 2A, B**). However, analysis of the markers of the G0/G1 or G1/S transition phases revealed a significant difference in the expression patterns of cell cycle regulators (**Figures 2C, D**). Cyclin D1 is a proto-oncogene that forms a complex with CDK-4 and -6 to regulate cell cycle progression at G1 and G1/S phases (50). The active form of cyclin D1, CDK4–CDK6 complex, phosphorylates and inactivates the tumor suppressor gene, retinoblastoma (*Rb*) (50, 51). Hence, high levels of cyclin D1, CDK4, and CDK6 are often observed in most patients with breast cancer (52, 53). Accordingly, we observed high levels of cyclin D1, CDK4, and CDK6 in MDA-MB-468 cells, which were significantly reduced by 2ME2 treatment in this study (**Figures 2C, D**). MDA-MB-231 cells also exhibited a similar response to 2ME2 in terms of Cyclin D1 regulation (**Supplementary Figures 2A, B**). Similarly, we analyzed the levels of the cell cycle inhibitor, p21, which inhibits the activity of CDK4 during the G1 phase of the cell cycle (54). p21 protein levels were higher in 2ME2-treated MDA-MB-468 cells than in the untreated control cells (**Figures 2C, D**). Our results demonstrate that 2ME2 induced cell cycle arrest in TNBC cells.

Cell death or apoptosis is poorly regulated in cancer cells, resulting in their uncontrolled growth (55). Here, we investigated whether 2ME2 induces apoptosis in MDA-MB-468 breast cancer cells. We used the Annexin V-FITC Apoptosis Detection Kit I with flow cytometry to evaluate apoptosis/cell death in 2ME2-treated TNBC cells. Cells treated with 2ME2 showed 9.2, 15.9, and 24.5% apoptotic death 24, 48, and 72 h after treatment, respectively (**Figures 2E, F**). To confirm this, we analyzed the regulators of apoptosis based on the expression levels of different apoptotic markers using immunoblotting analysis. Notably, 2ME2 treatment significantly increased the levels of cleaved PARP, cleaved caspase 3 and BAK but decreased the levels of Bcl-2 (**Figures 2G, H**). Finally, we investigated the effect of 2ME2 on Bak and cleaved caspase-3 expression using MDA-MB-231 cells, which demonstrated the activation of these apoptotic markers at all tested time points (**Supplementary Figures 2C, D**). This finding indicates that 2ME2 acts as an anti-cancer agent by inducing intrinsic mediated apoptosis in TNBC cells.

### 3.4 Estrogen metabolite 2ME2 disrupts global miRNome profiling

**Figure 3:**
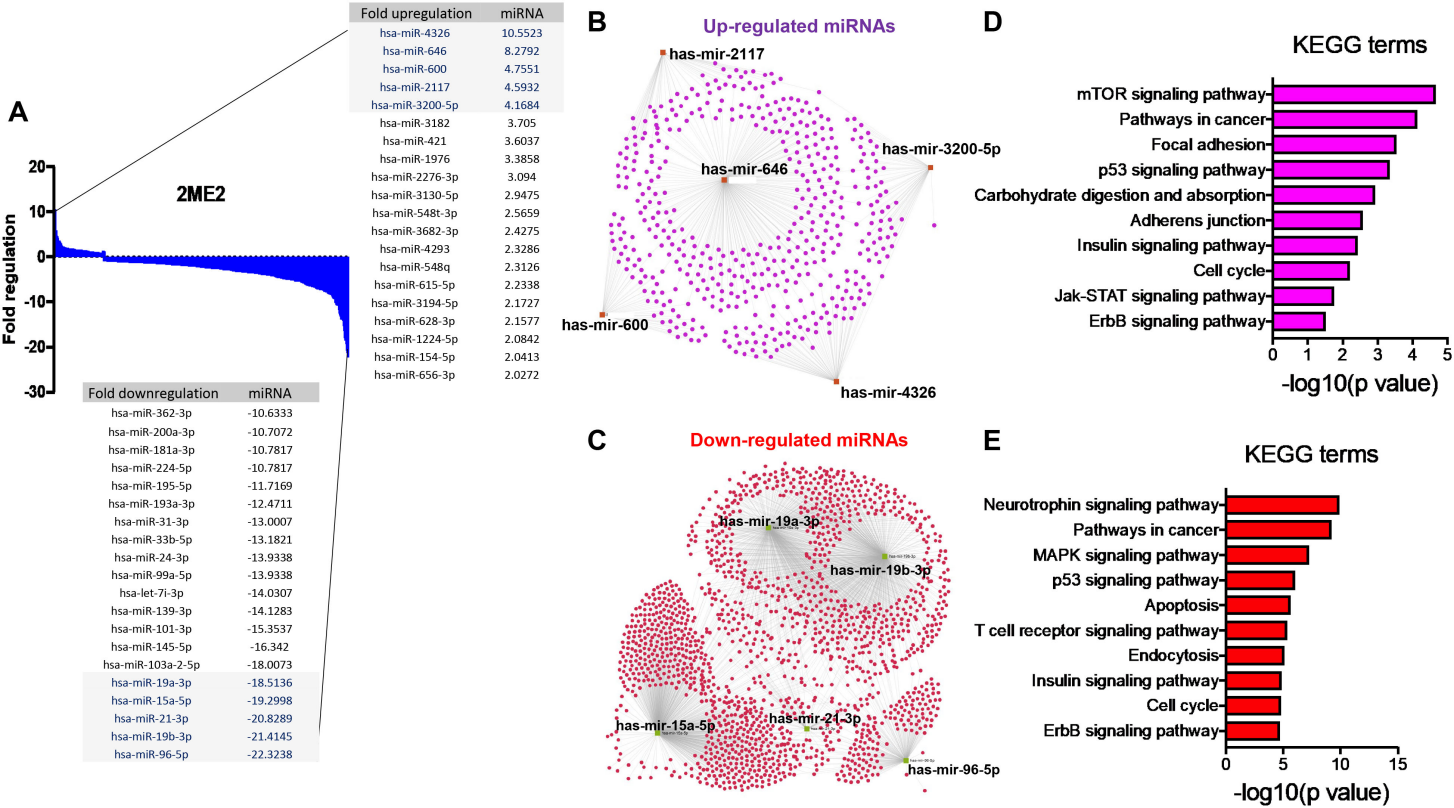
2ME2 influences global miRNome profile and network analysis identifies target genes. **(A)** Human miRNome profiling with 2ME2 treated MDA-MB-468 cells identified differentially regulated miRNAs. The 20 most upregulated and downregulated miRNAs were identified all which had a greater than 2 fold difference. **(B)** The top five upregulated miRNAs depicted in red with their target genes highlighted in purple. **(C)** The top five downregulated miRNAs are also depicted in green with their respective target genes highlighted in red. **(D, E)** The top 5 up and down-regulated miRNAs influences different signaling pathways via KEGG pathway analysis.

The specific effects of 2ME2 on TNBC miRNomes remain unknown. Therefore, in this study, we explored the miRNA disruptions. MDA-MB-468 cells treated with 2ME2 (5 µg for 24 h) were subjected to miRNome analysis. Over 1,000 mature miRNAs were studied using the miScript miRNA PCR array (miRNome V16). We identified the top 20 most upregulated and downregulated miRNAs (**Figure 3A**). We validated the differential expression of top 5 up- and down-regulated miRNAs using RTPCR in 2ME2 treated MDA-MB-468 (**Supplementary Figures 3A, B**) and MDA-MB-231 cells (**Supplementary Figures 3C, D**). Here, we aimed to identify the target genes of both the upregulated and downregulated miRNAs. In silico analysis was performed using the miRNet database to identify the miRNA target genes (**Figures 3B, C**). Moreover, we used the miRNet database to identify the signaling pathways associated with the genes altered by 2ME2 through miRNAs. KEGG pathway analysis demonstrated that the upregulated miRNA target genes were mainly involved in mTOR signaling, cancer-related, and p53 signaling pathways (**Figure 3D**). Downregulated miRNA target genes were also involved in cancer-related, p53 signaling, mitogen-activated protein kinase (MAPK) signaling, and apoptosis pathways (**Figure 3E**). Further, the impact of some of the differentially regulated miRNAs on overall survival was assessed using Kmplot and UALCAN, which demonstrated that the miRNAs upregulated (hsa-miR-600, hsa-miR-2117 & hsa-miR-3200) and downregulated miRNAs (hsa-miR-96, hsa-miR-19a & hsa-miR-19b-2) by 2ME2 increased survival of triple negative breast cancer patients (**Supplementary Figures 4A, B**). These data demonstrate that 2ME2 effectively alters miRNome.

### 3.5 miRNA target genes are upregulated in human breast cancer

**Figure 4:**
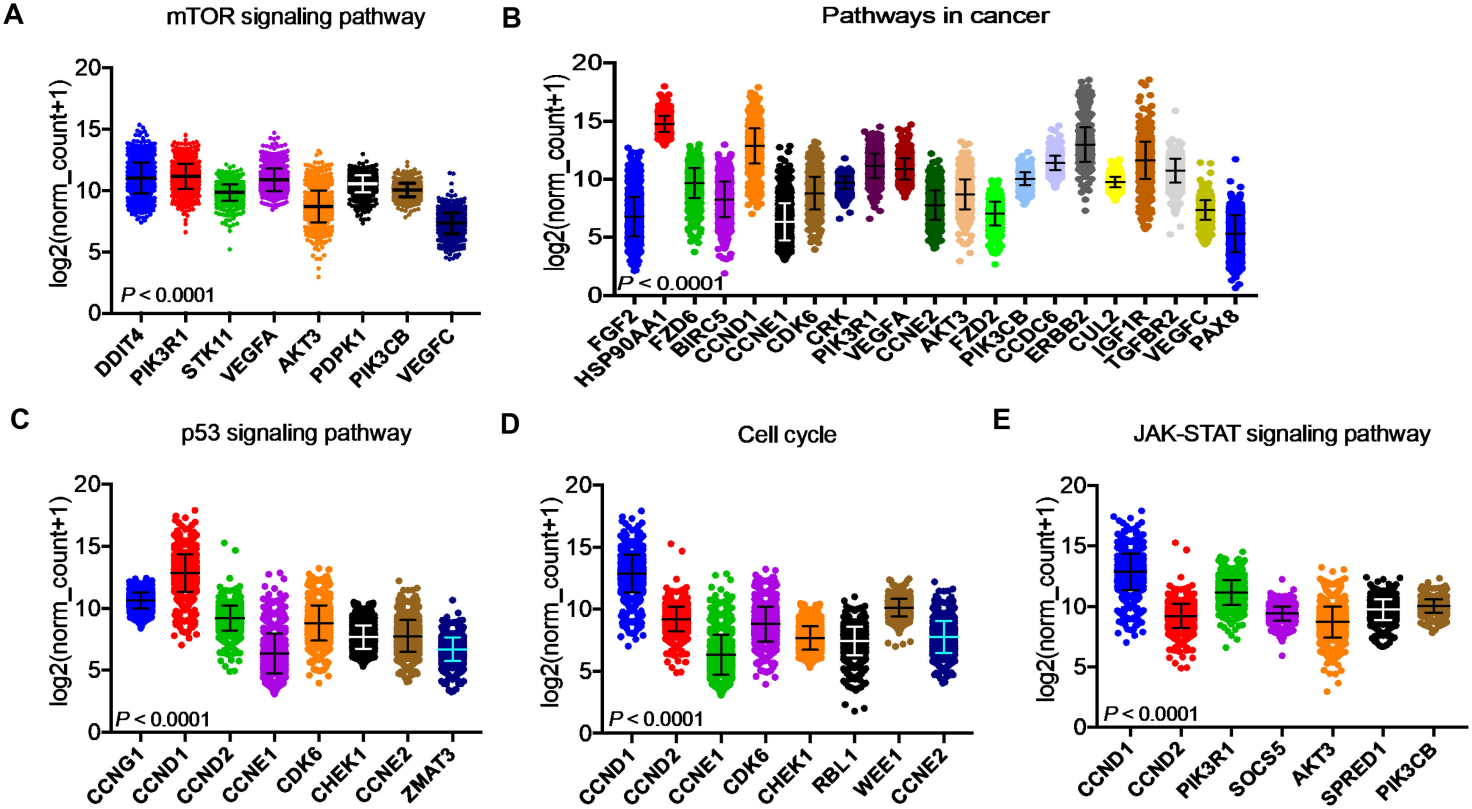
Up-regulated miRNA DEG targets. **(A-E)** miRNA target genes upregulated in breast cancers following 2ME2 treatment identified using Network analysis. KEGG analysis identified top miRNA direct target genes that are involved in different pathways. **(A)** mTOR signaling pathway, **(B)** pathways in cancer, **(C)** p53 signaling pathway, **(D)** cell cycle, and **(E)** JAK-STAT signaling pathway.

**Figure 5:**
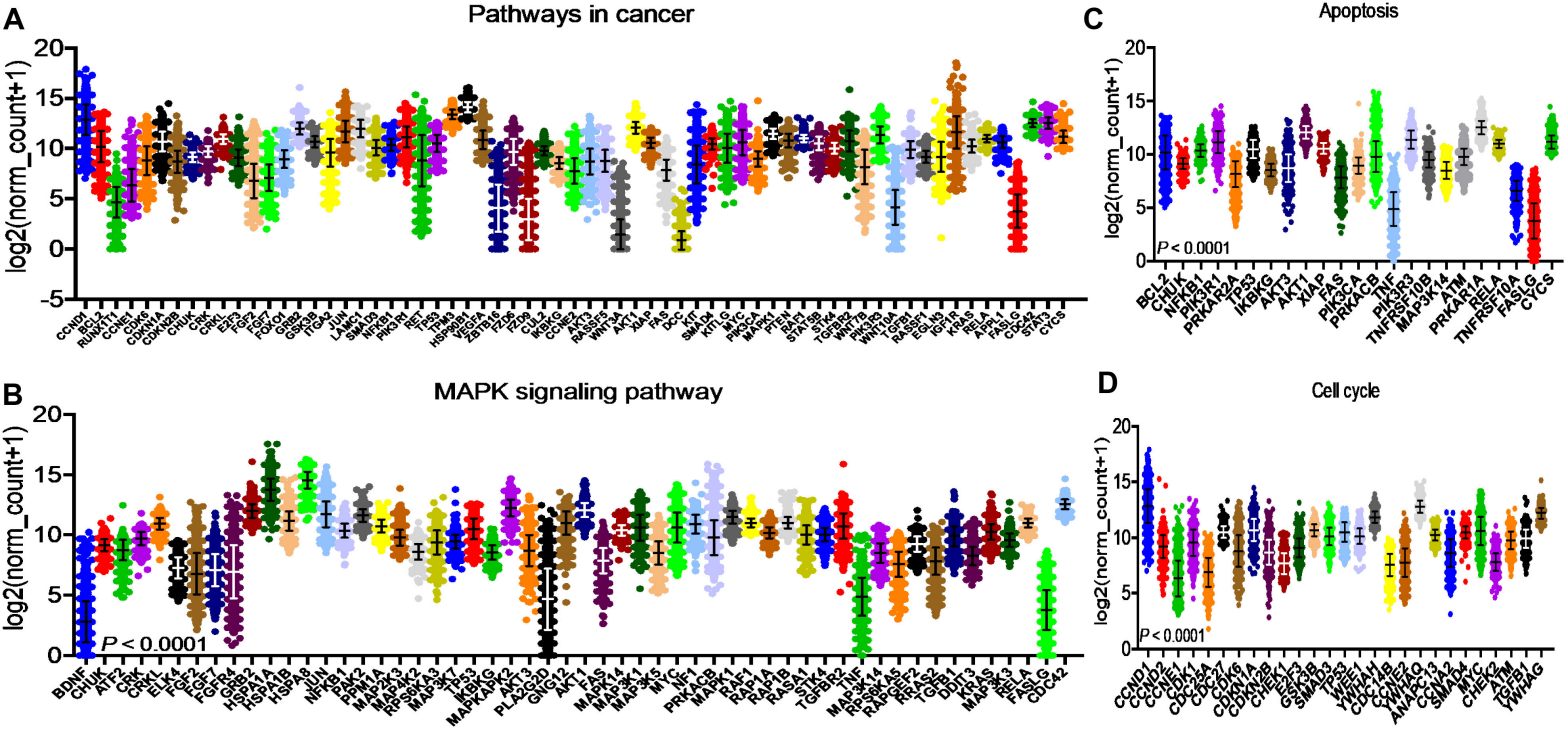
Down-regulated miRNA DEG targets. **(A-D)** miRNA target genes down regulated in breast cancers following 2ME2 treatment identified using Network analysis. KEGG analysis identified top miRNA direct target genes that are involved in different pathways. **(A)** pathways in cancer, **(B)** MAPK signaling pathway, **(C)** Apoptosis, and **(D)** cell cycle.

**Figure 6:**
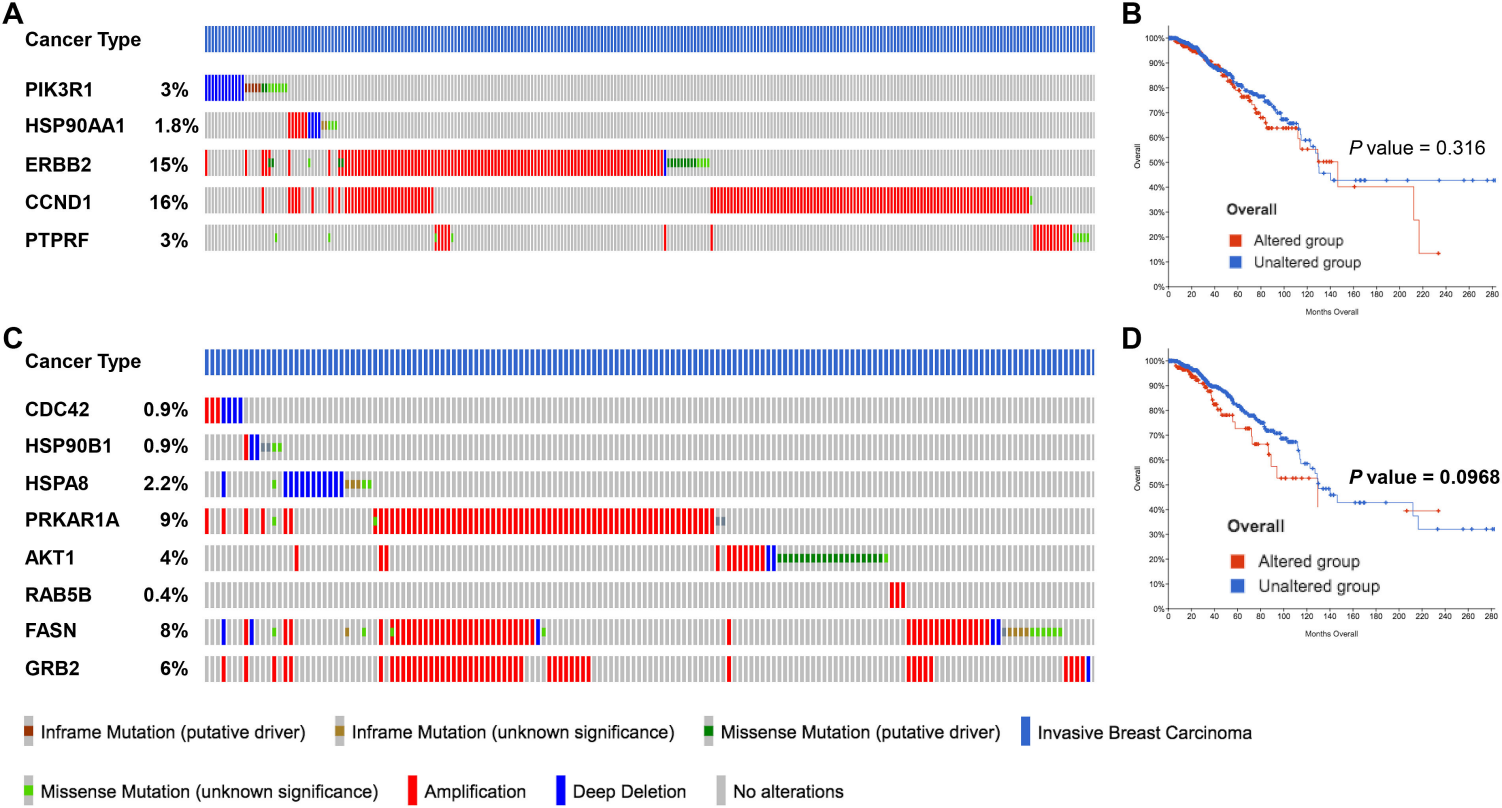
miRNA target genes altered by 2ME2 treatment are amplified in breast cancer patients. **(A, B)** Five target genes of up-regulated miRNA in 2ME2 treatment, **(A)** Oncoprint represents genetic alterations such as inframe mutations (putative driver and unknown significance), missense mutation (putative driver and unknown significance), amplification and deep deletion of breast cancer tumor samples. **(B)** KMPlot analysis shows cumulatively breast cancer patient samples that have high expression of miRNA target genes that are associated with overall survival. **(C, D)** Eight target gene of down-regulated miRNA in 2ME2 treatment, **(C)** Oncoprint represents genetic alterations such as inframe mutations (putative driver and unknown significance), missense mutation (putative driver and unknown significance), amplification and deep deletion of breast cancer tumor samples. **(D)** KMPlot analysis shows cumulatively breast cancer patient samples that have altered expression of miRNA target genes that are associated with overall survival. In KMPlot, unaltered means that there were no gene alterations in the queried genes within the selected profile of 2ME2 treated samples. Altered means that there was at least one gene altered in the queried genes within the selected profile of 2ME2 treated samples.

After identifying the miRNA target genes using the miRNet database, we uploaded the target genes to the Xena browser to study their expression patterns. Xena browser database enabled the exploration of gene expression directly using the human samples profiled from TCGA database. Then, the expression patterns of miRNA target genes from mTOR signaling, cancer signaling, focal adhesion, p53 signaling, carbohydrate digestion and absorption, insulin signaling, cell cycle, JAK-STAT, ErBb signaling, and adherens junction pathways were plotted (**Figures 4A–E** and **Supplementary Figures 5A–E**). Downregulated miRNA target genes were associated with neurotrophin signaling, cancer signaling, p53 signaling, MAPK signaling, apoptosis, T cell receptor signaling, endocytosis, insulin signaling, cell cycle, and ErBb signaling pathways (**Figures 5A–D** and **Supplementary Figures 6A–F**). *PIK3R1*, *HSP90AA1*, *ERBB2*, *CCND1*, and *PTPRF* were the target genes of the top five upregulated miRNAs (**Figures 6A**, **B**). Meanwhile, *CDC42*, *HSP90B1*, *CCND1*, *HSPA8*, *PRKAR1A*, *AKT1*, *RAB5B*, *FASN*, and *GRB2* were the target genes of the top downregulated miRNAs (**Figure 6C**). The expression of these genes were validated using RTqPCR. In general the all the target genes of up-regulated miRNAs were down regulated significantly by 2ME2 treatment in both TNBC cell lines. Interestingly, we also observed that some of the target genes of the down-regulated miRNAs were significantly upregulated (**Supplementary Figures 7A-D**). These genes have been shown to influence overall survival of breast cancer patients. When we analyzed combined effect of these up- and down-regulated genes on overall survival, we observed the expected trends but they were not statistically significant. (**Figure 6D**).

### 3.6 Signal transduction pathways altered by *2ME2* were identified using Reactome

**Figure 7:**
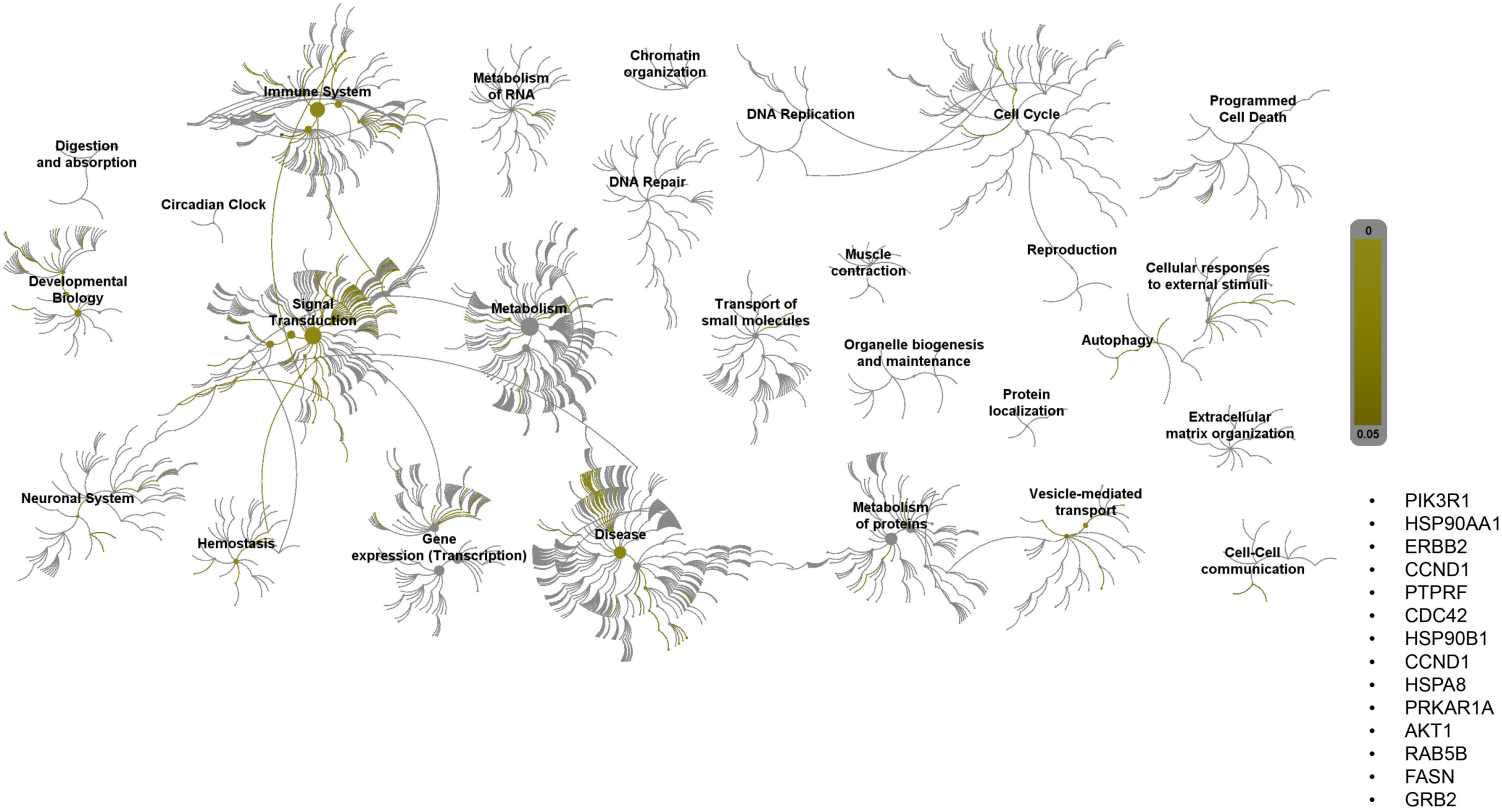
Reactome pathway-topology analysis of miRNA target genes. Reactome pathway analysis of the genes identified by KEGG term analysis. Target genes of miRNAs altered by 2ME2 treatment influenced various cellular pathways. Over-represented pathways are highlighted in yellow. All overexpressed pathways are from gene lists of formerly annotated and published signatures.

Pathway topology analysis of miRNA target genes was performed using the Reactome pathway identifier database. In silico analysis enabled the identification of the differentially expressed genes mapped to different pathways. Interestingly, 2ME2 treatment led to the activation of various pathways, including the development, signal transduction, immune system, and vehicle-mediated transport pathways (**Figure 7**).

## 4 Discussion

2ME2 is an endogenous metabolite of estrogen. It is produced when 17 β-estradiol undergoes hydroxylation by cytochrome P450 1A1 to produce 2-hydroxyestradiol, followed by methylation by catechol-O-methyltransferase to produce 2-ME2. Compared with 17 β-estradiol, 2ME2 has a very low affinity (<500 fold) for ERα (56). Here, we demonstrated the anti-cancer effects of 2ME2 against TNBC. The action of 2ME2 on normal and breast cancer cells is interesting because the production of 2ME2 from 2-hydroxyestradiol via O-methylation is associated with a reduced risk of breast cancer (12, 57). Metabolic pathways involved in the production of 4-hydroxyestradiol and 16α-hydroxyestrone increase the risk of breast and endometrial cancers (58, 59). Interestingly, our data demonstrated that estrogen metabolite 2ME2 exerts mild apoptotic effects on normal mammary cells in culture. In the past few years, many studies have focused on 2ME2 to better understand its anti-cancer properties and unique biological effects. Cellular and molecular action mechanisms of this estrogen metabolite indicate its potential as a chemotherapeutic drug for different types of human cancer (60, 61). Pharmacological concentrations of 2ME2 have been reported to exert strong anti-proliferative, anti-angiogenic, and apoptotic effects in several cancer models, leading to many phase I and II clinical trials (62). 2ME2 acts directly on the vascular endothelial cells, inhibiting their proliferation and migration and decreasing their drug resistance (63). In addition to its anti-angiogenic properties, we found that 2ME2 also exerts anti-proliferative effects and inhibits metastasis by suppressing proliferative markers, such as AKT and mTOR, and by reducing the migration, invasion, and colony formation of TNBC cell lines. Everolimus, an AKT/mTOR inhibitor, can only be used for the treatment of hormone receptor-positive and human epidermal growth factor receptor 2-negative metastatic breast cancer (64); however, 2ME2, which acted as an AKT/mTOR inhibitor in this study, can be used as a promising drug for TNBC treatment. Additionally, 2ME2 acts synergistically with other chemotherapeutic drugs to target breast cancer cells. When combined with doxorubicin, 2ME2 increases the sensitivity of doxorubicin-resistant breast cancer cells to doxorubicin by increasing the apoptosis and cell cycle arrest at the G1 phase in MCF-7 cells (65). When combined with paclitaxel (Taxol), 2ME2 increases centrosome decluttering and kills the MCF-7, MDA-MB-231, and SUM-149 breast cancer cells (66).

Various anti-cancer activities of 2ME2, including cell cycle regulation, have been proposed for different cancers. It acts on pancreatic cancer by prolonging the cell cycle events during the S phase and inducing apoptosis (67). In TNBC cell lines, 2ME2 treatment arrests the cells in the S-phase, in which DNA replication, single strand breaks (SSBs), and repair occurs. PARP, which repairs SSBs (68) is inhibited by 2ME2, thereby increasing the apoptosis and inhibiting the excessive growth of TNBC cell lines. Various mechanisms of action of 2ME2 have been reported for the growth control of different cancers, including breast cancer. To the best of our knowledge, this is the first study to reveal that 2ME2 treatment affects the global miRNA profile in TNBC. miRNAs play key roles in human diseases, particularly cancer. miRNAs are differentially expressed, acting as either oncogenes or tumor suppressors in cancer cells (35). miRNAs regulate the functions of many genes, and a single miRNA can control the functions of different genes. We identified many signaling pathways that were upregulated or downregulated in 2ME2-treated MDA-MB-468 TNBC cell line. Analysis of the gene targets of the upregulated and downregulated miRNAs revealed that these genes were mostly involved in signal transduction and immune pathways. Cyclin D1 is a cell cycle regulator that is overexpressed in breast cancer (69). Here, cyclin D1 and its regulator, CDK6, were found to be the top target genes of both upregulated and downregulated miRNAs. It is well-known that miRNAs regulate gene expression both negatively and positively by direct inhibition or indirectly through other regulators of gene expression. Our data indicates that the expression of cyclin D1 and CDK6 are tightly regulated by various miRNAs.

Mechanisms underlying the anti-cancer effects of 2ME2 are complex, warranting further investigation. A limitation of the current study is that the specific molecular mechanisms are not identified. In this study, miRNome analysis revealed many pathways are affected by 2ME2 treatment. Our findings also revealed the miRNAs control key pathways in cancer and their target genes. Further, our results are expected to provide the basis to further elucidate the specific mechanisms by which 2ME2 inhibits breast cancer and also could lead to the development of 2ME2 as a promising therapeutic drug for TNBC.

## Data Availability

The original contributions presented in the study are included in the article/**Supplementary Material**. Further inquiries can be directed to the corresponding authors.
