# Supplementary material for: 2-methoxyestradiol inhibits the malignant behavior of triple negative breast cancer cells by altering their miRNome
Source: Front Oncol. 2024 Sep 12;14:1371792. doi: 10.3389/fonc.2024.1371792 (PMC11424607; doi:10.3389/fonc.2024.1371792)
Supplement: Supplementary file 1 [file DataSheet1.pdf]

Supplementary Results

Supplementary Figure 1A

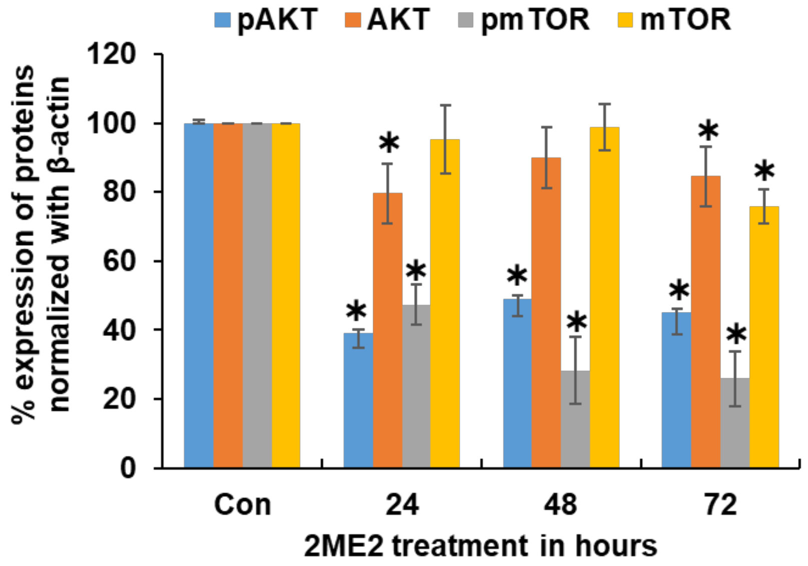

Supplementary Figure 1B

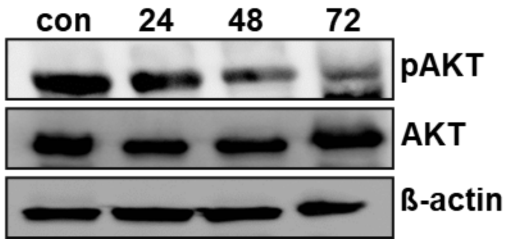

Supplementary Figure 1C

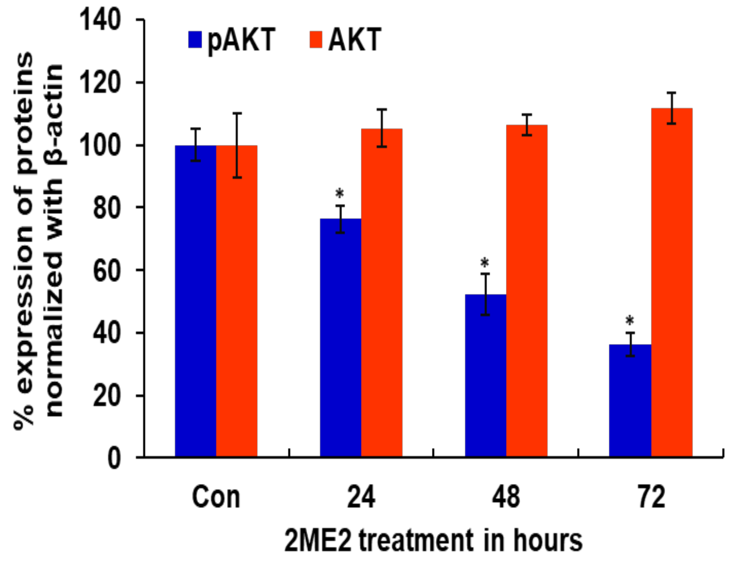

Supplementary Figure 1D

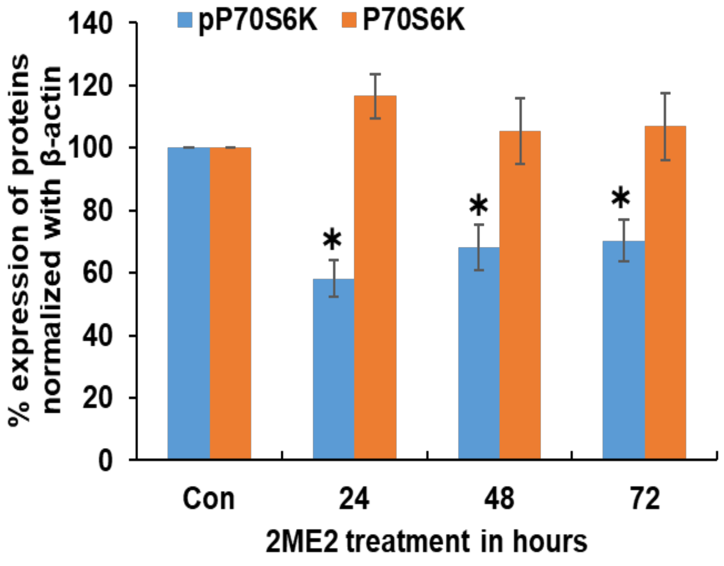

Supplementary Figure 1E

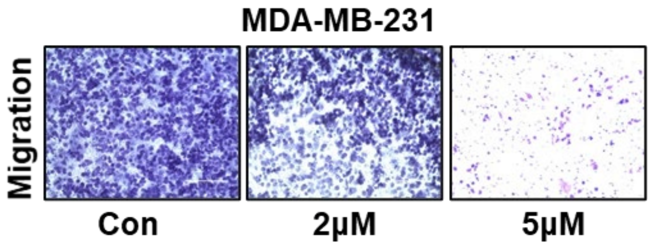

Supplementary Figure 1F

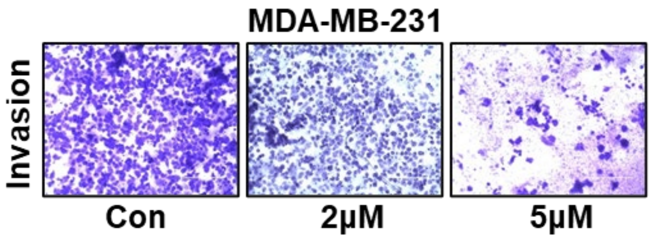

Supplementary Figure 1G

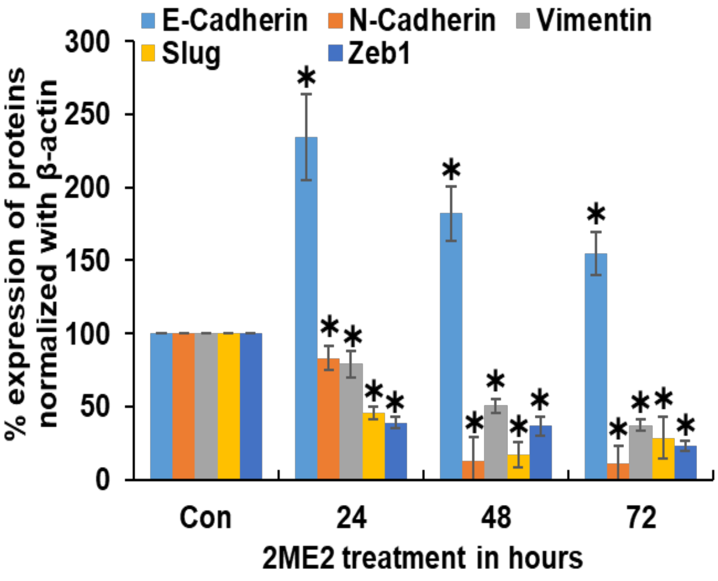

Supplementary Figure 1H

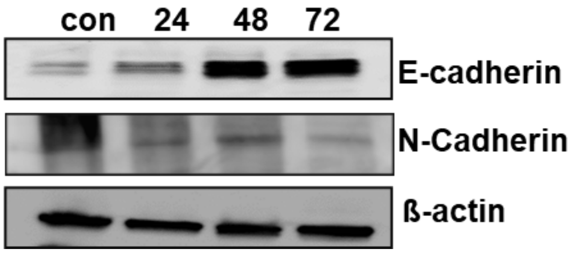

Supplementary Figure 1I

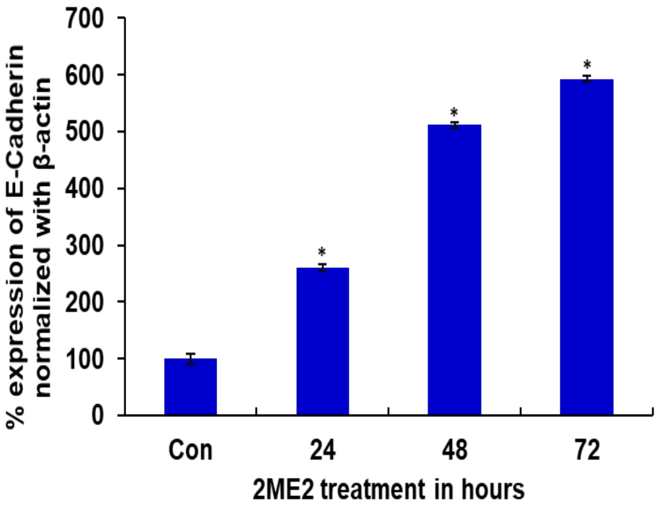

Supplementary Figure 1J

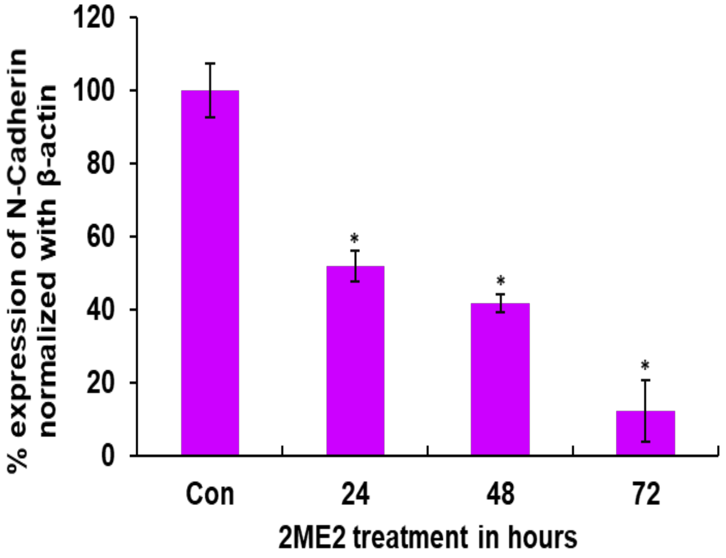

Supplementary Figure 1: The data presented in supplementary figure 1A is the densitometric analysis of Western blot data represented in Figure 1B.

Expression level and densitometric analysis of pAKT and AKT in MDA-MB-231 cells at different time points of treatment with 5μM of 2ME2 (Supplementary figure 1B and C).

Effect of 2ME2 on migratory and invasive capacity of MDA-MB-231 cells (Supplementary figure 1E and F).

The data presented in supplementary figure 1G is the densitometric analysis of Western blot data represented in Figure 1J.

E-Cadherin and N-Cadherin expression in 2ME2 treated MDA-MB-231 cells at different time points (Supplementary figure 1H) and densitometric analysis of these markers (Supplementary figure 1I and J) respectively.

A value of $p < 0.05$ was considered statistically significant.

Supplementary Figure 2A

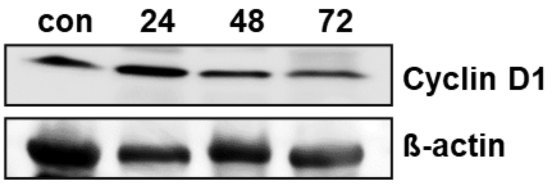

Supplementary Figure 2B

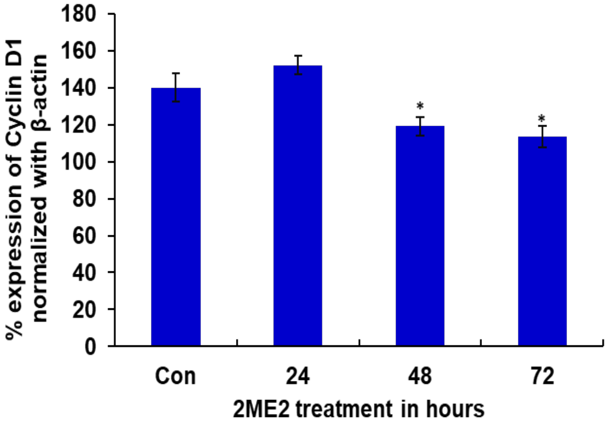

Supplementary Figure 2C

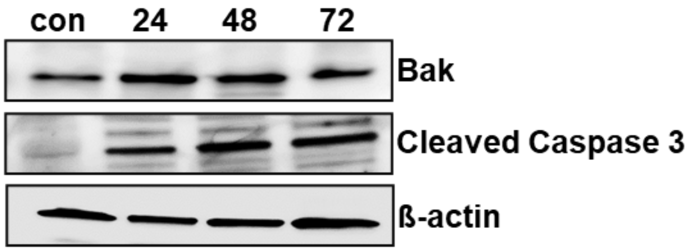

Supplementary Figure 2D

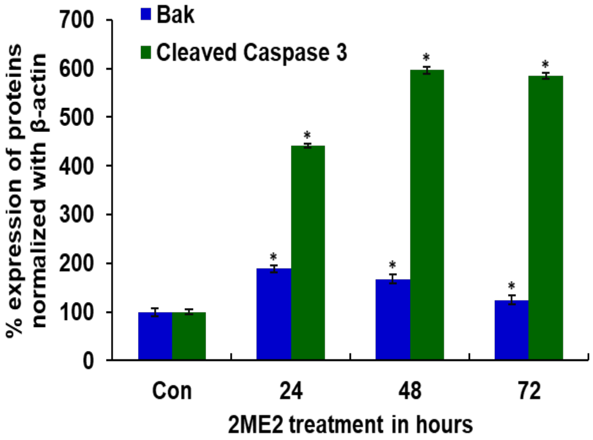

Supplementary Figure 2: The data presented in supplementary Figure 2A and B are the cell cycle regulator Cyclin D1 and internal control beta actin were assessed in 2ME2 treated MDA-MB-231 cells at 24, 48 and 72h using Western blot and densitometric analysis. Expression levels and densitometric analysis of Bak and cleaved caspase-3 in 2ME2 treated MDA-MB-231 cells at different time points (Supplementary Figure 2C and D) respectively. A value of $p < 0.05$ was considered statistically significant.

Supplementary Figure 3A:

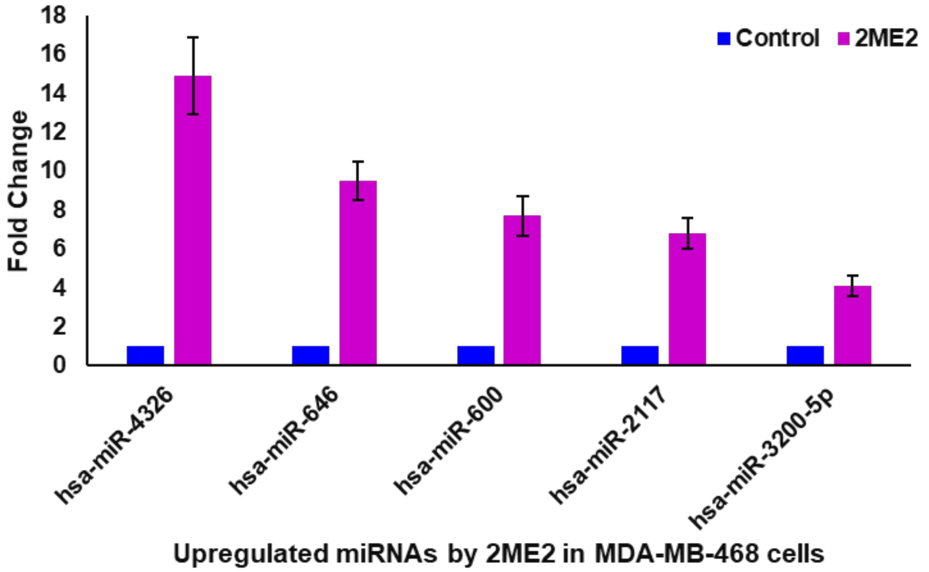

Supplementary Figure 3B:

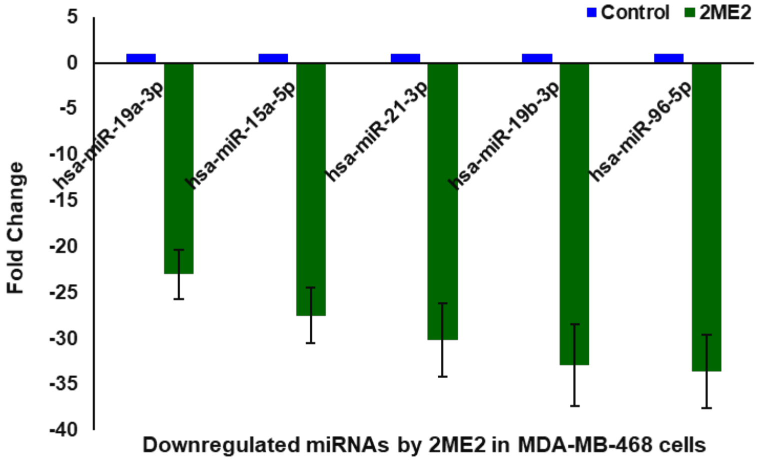

Supplementary Figure 3C:

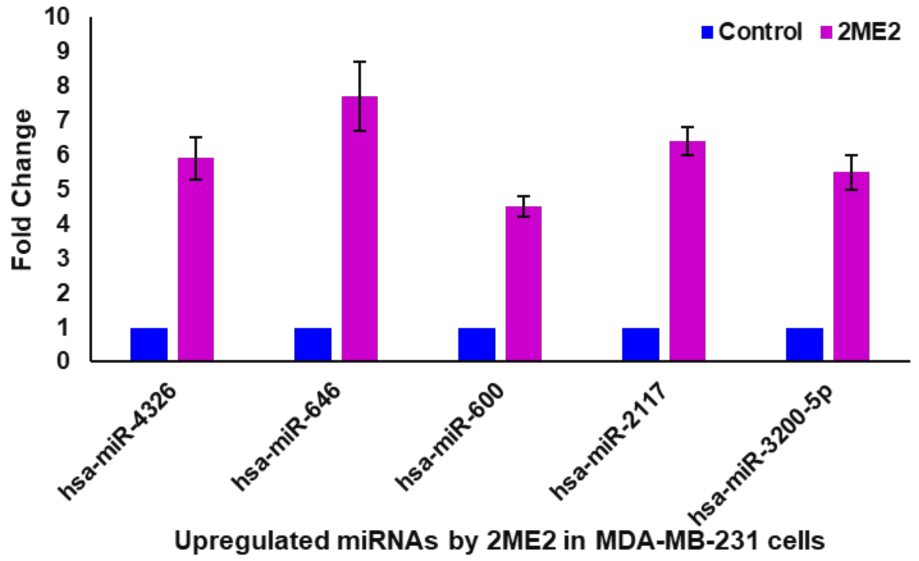

Supplementary Figure 3D:

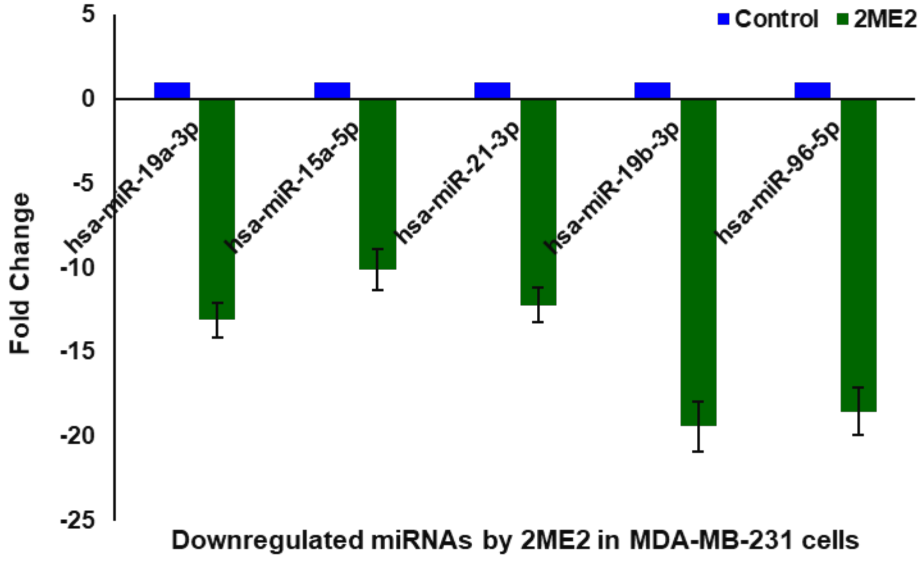

Supplementary Figure 3: A & B: RT-PCR analysis were performed to confirm the effect of 2ME2 on top 5 upregulated and downregulated miRNAs in MDA-MB-468 cells and **C & D:** RT-PCR analysis were performed to confirm the effect of 2ME2 on top 5 upregulated and downregulated miRNAs in MDA-MB-231 cells using the specific primers sequences mentioned in table 2.

Supplementary Figure 4A

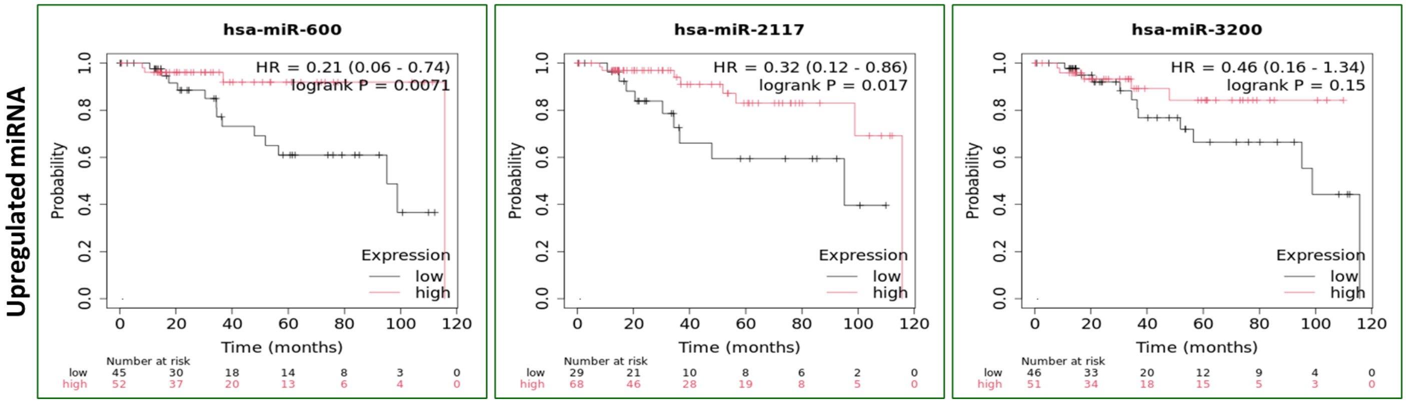

Supplementary Figure 4B

Downregulated miRNA has-mir-96

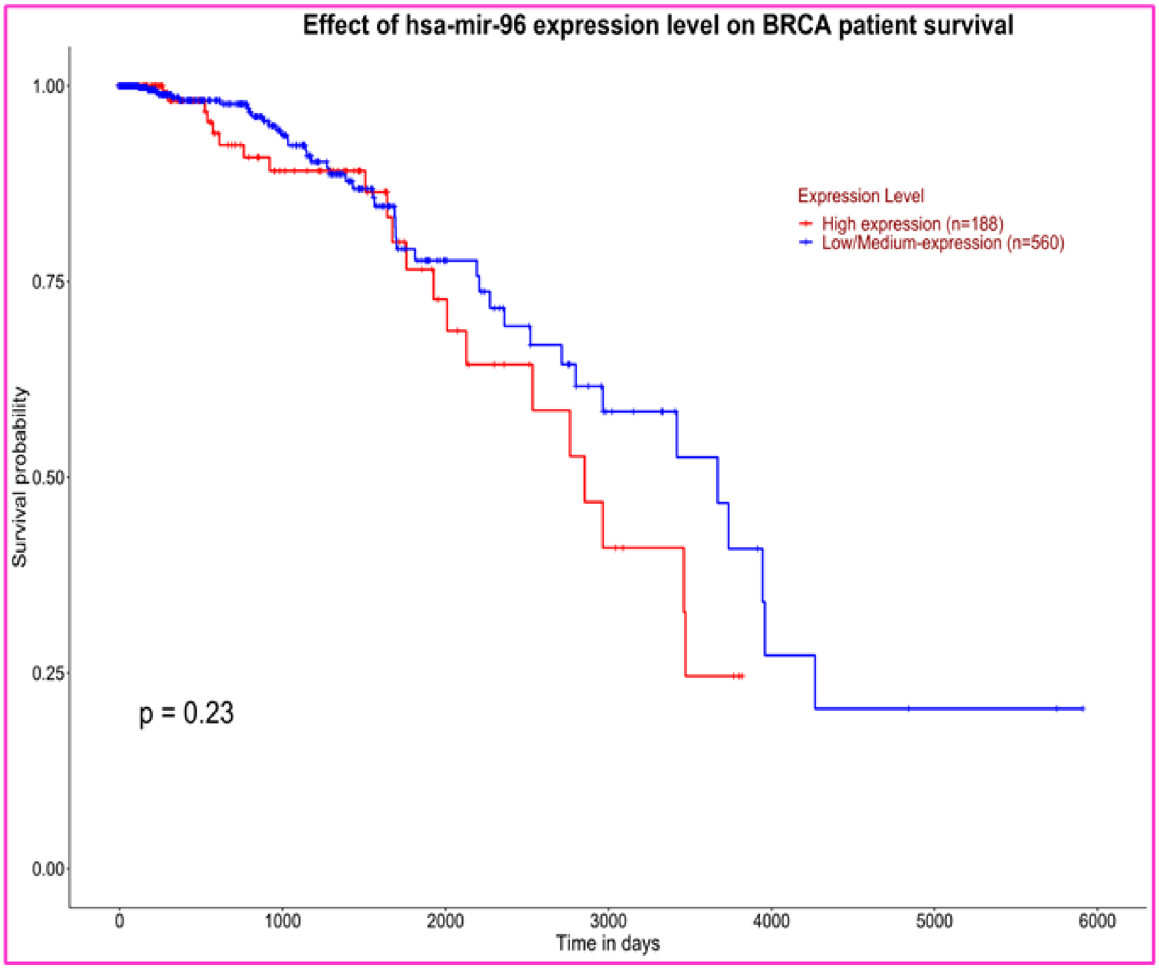

Downregulated miRNA has-mir-19a

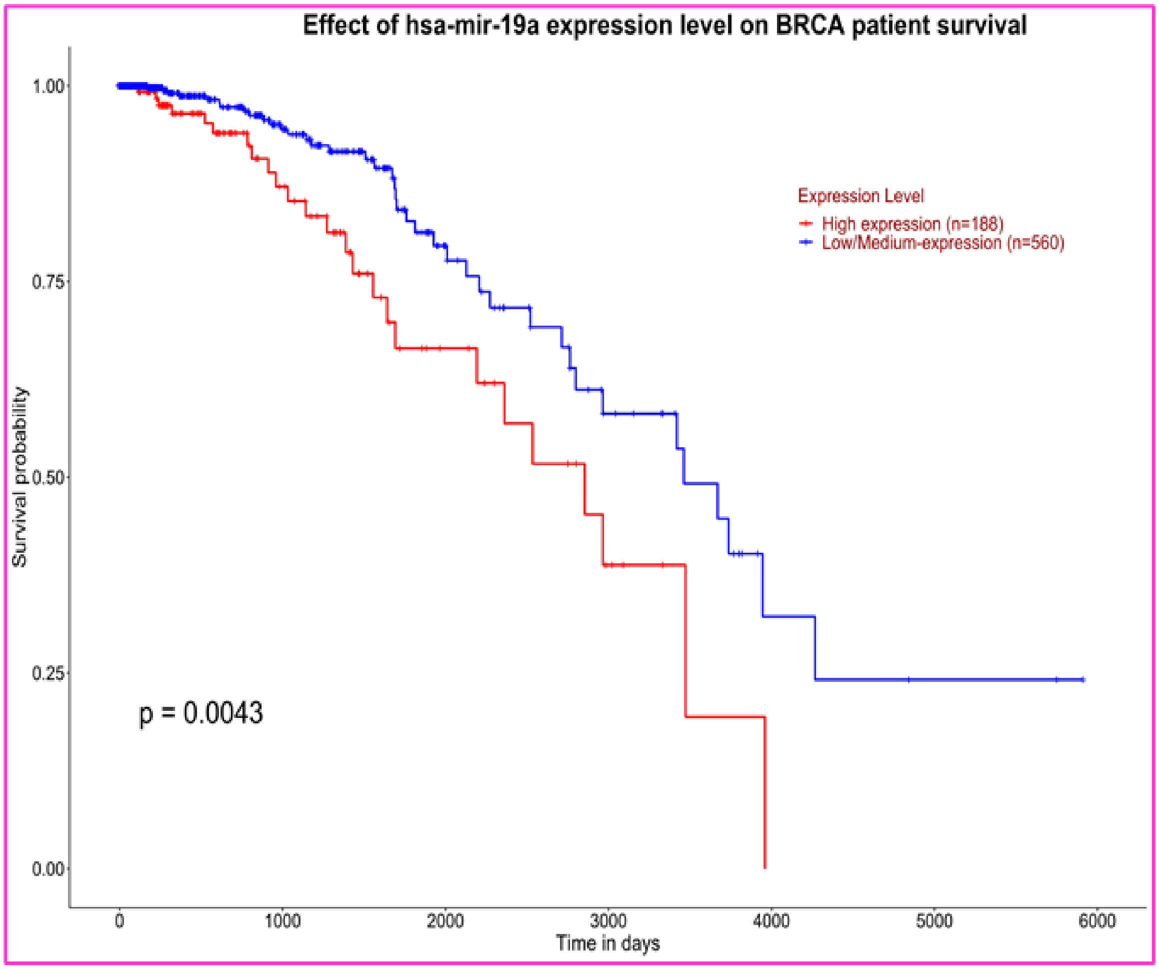

Downregulated miRNA has-mir-19b-2

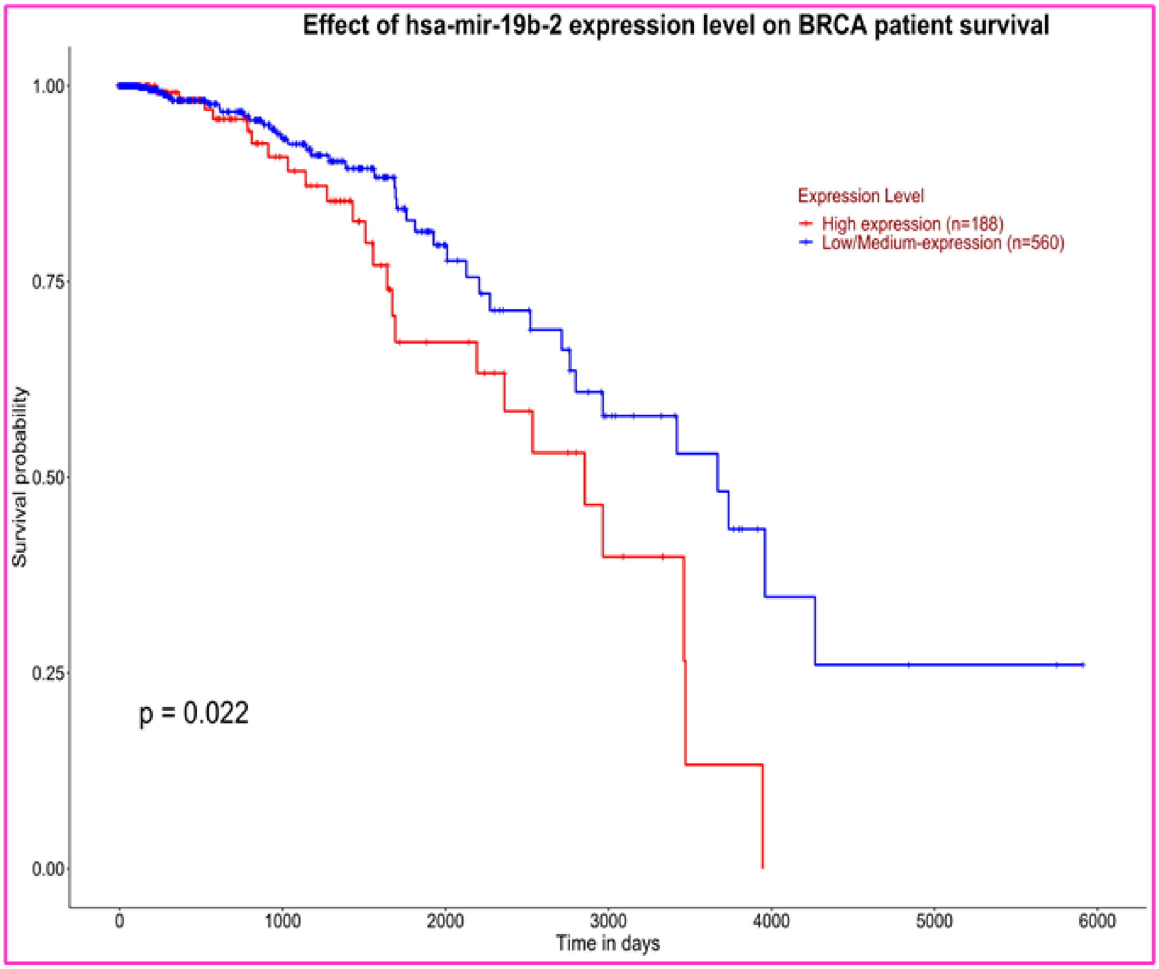

Supplementary Figure 4: Survival analysis of miRNAs altered by 2ME2 treatment are amplified in breast cancer patients.

(A): Three up-regulated miRNA in 2ME2 treatment subjected to KMPlot analysis which shows cumulatively breast cancer patient samples that have altered expression of miRNAs (hsa-miR-600, and hsa-miR-2117 and hsa-miR-3200) that are associated with overall survival. (B): Three down-regulated miRNA in 2ME2 treatment subjected to UALCAN survival analysis which shows cumulatively breast cancer patient samples that have altered expression of miRNAs (hsa-mir-96, hsa-mir-19a and hsa-mir-19b-2) that are associated with overall survival.

Supplementary Figure 5A

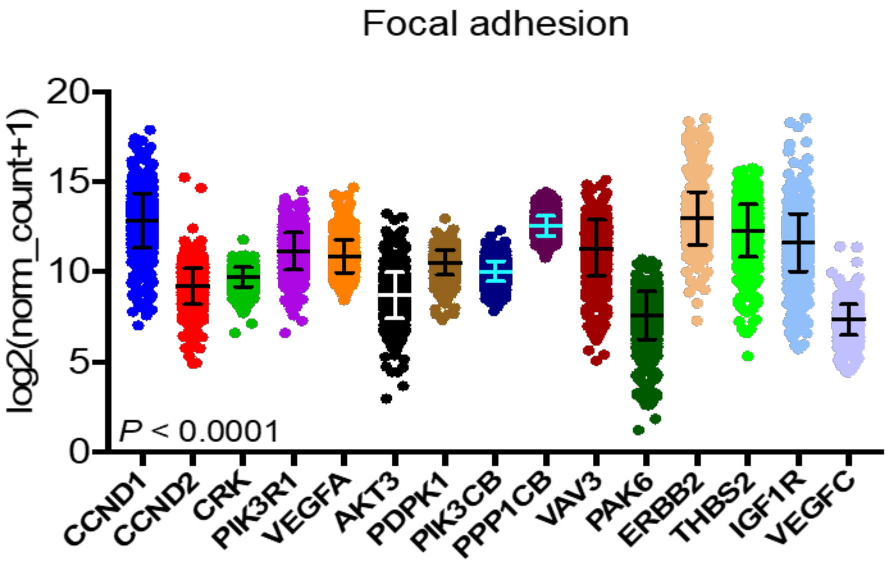

Supplementary Figure 5B

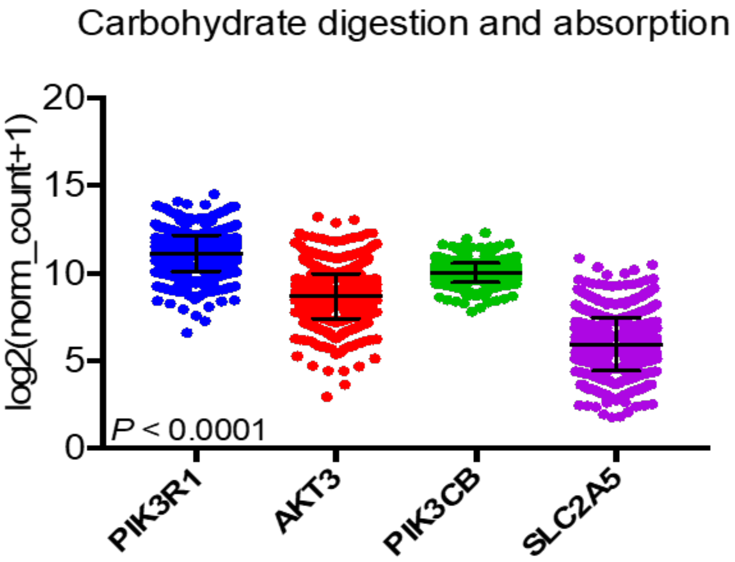

Supplementary Figure 5C

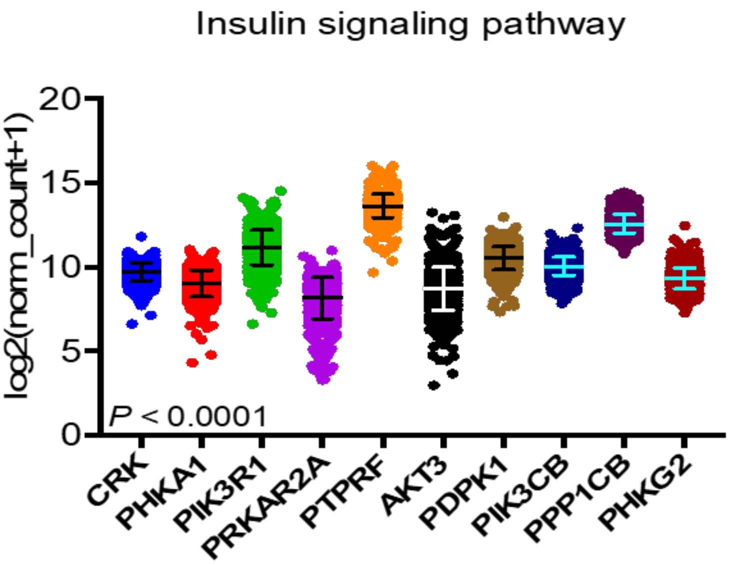

Supplementary Figure 5D

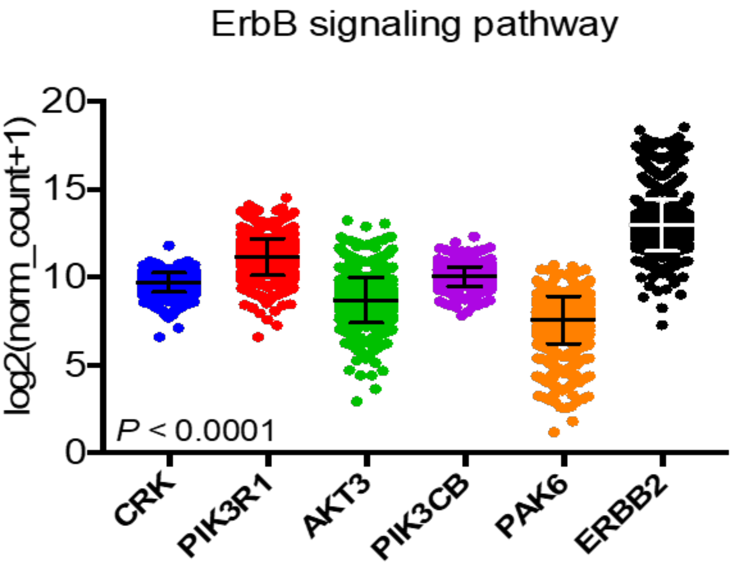

Supplementary Figure 5E

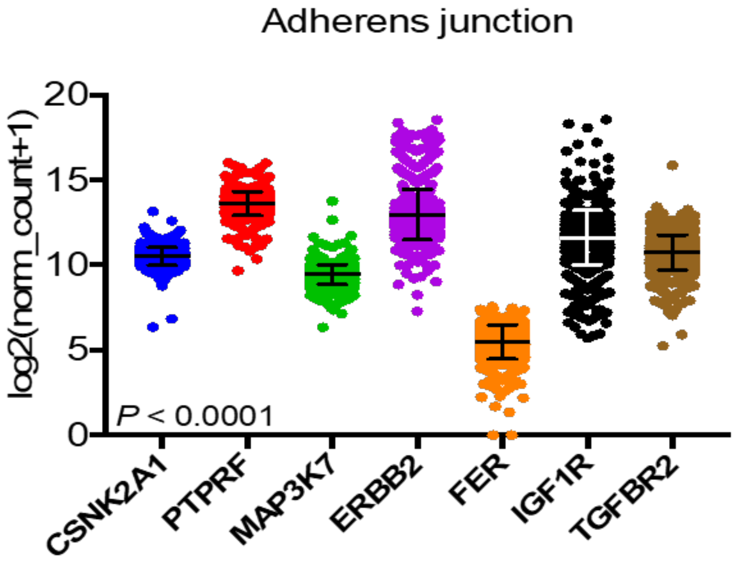

Supplementary Figure 5: Up-regulated miRNA DEG targets.

A-E) miRNA target genes upregulated in breast cancers following 2ME2 treatment identified using Network analysis. KEGG analysis identified top miRNA direct target genes that are involved in different pathways. A) focal adhesion, B) carbohydrate digestion and absorption, C) insulin signaling pathway, D) ErbB signaling pathway and E) Adherens junction.

Supplementary Figure 6A

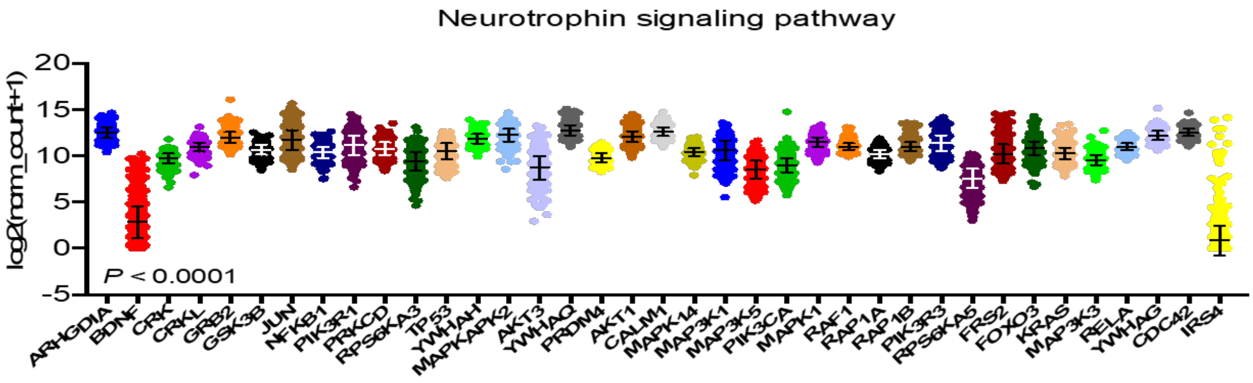

Supplementary Figure 6B

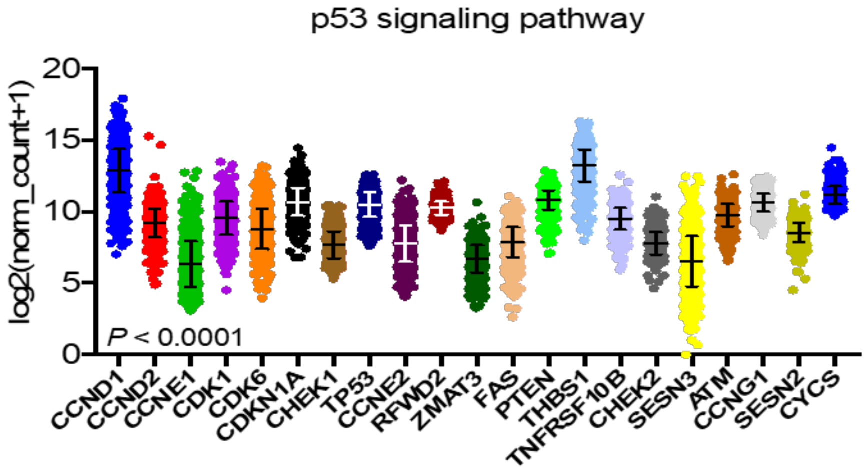

Supplementary Figure 6C

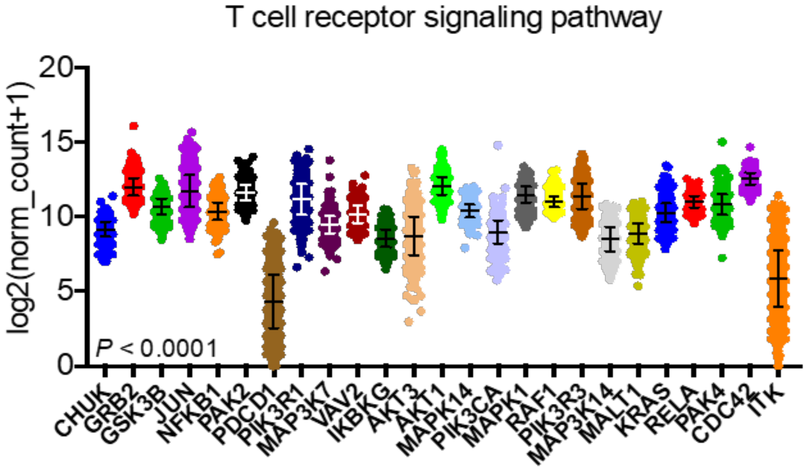

Supplementary Figure 6D

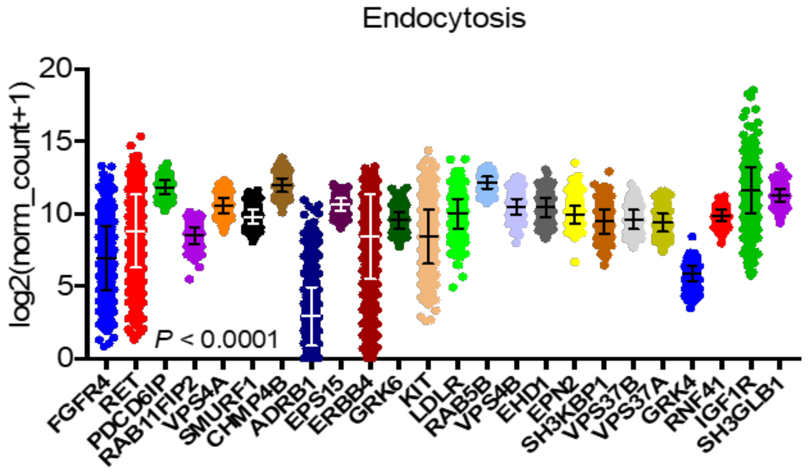

Supplementary Figure 6E

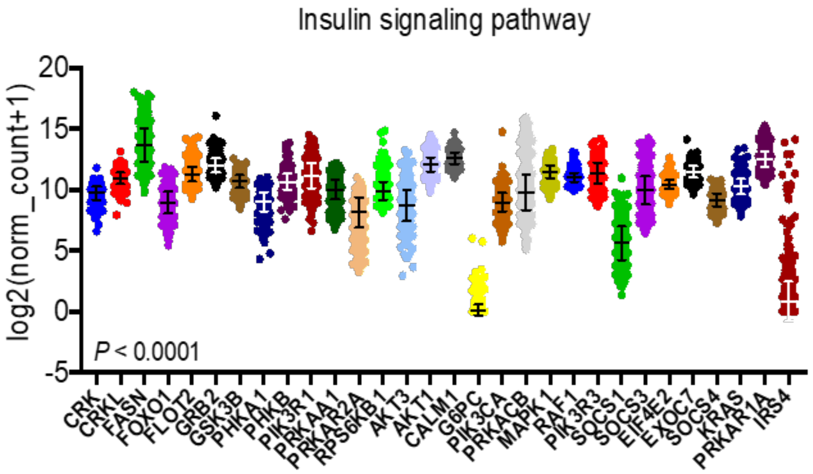

Supplementary Figure 6F

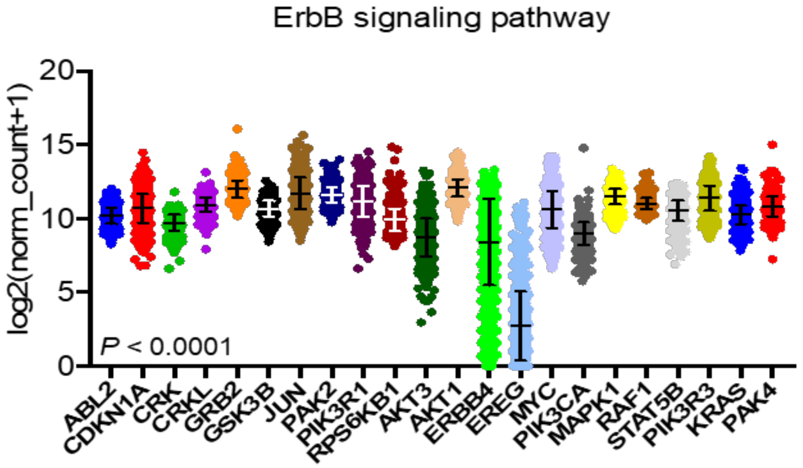

Supplementary Figure 6: Down-regulated miRNA DEG targets.

A-F) miRNA target genes down regulated in breast cancers following 2ME2 treatment identified using Network analysis. KEGG analysis identified top miRNA direct target genes that are involved in different pathways. A) neurotrophin signaling pathway, B) p53 signaling pathway, C) T-cell receptor signaling pathway, D) Endocytosis, E) insulin signaling pathway, F) ErbB signaling pathway.

Supplementary Figure 7A

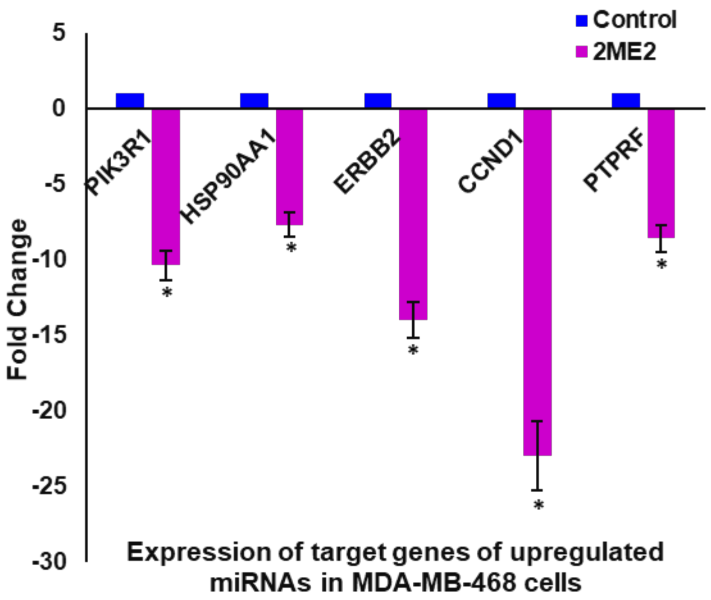

Supplementary Figure 7B

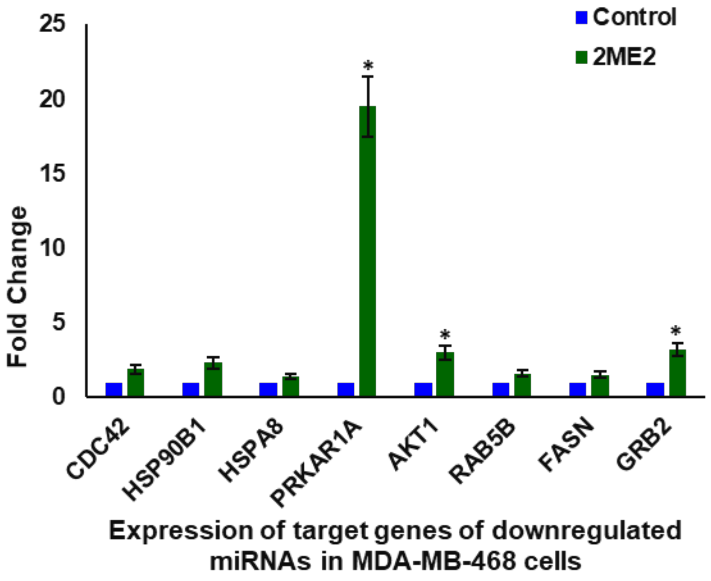

Supplementary Figure 7C

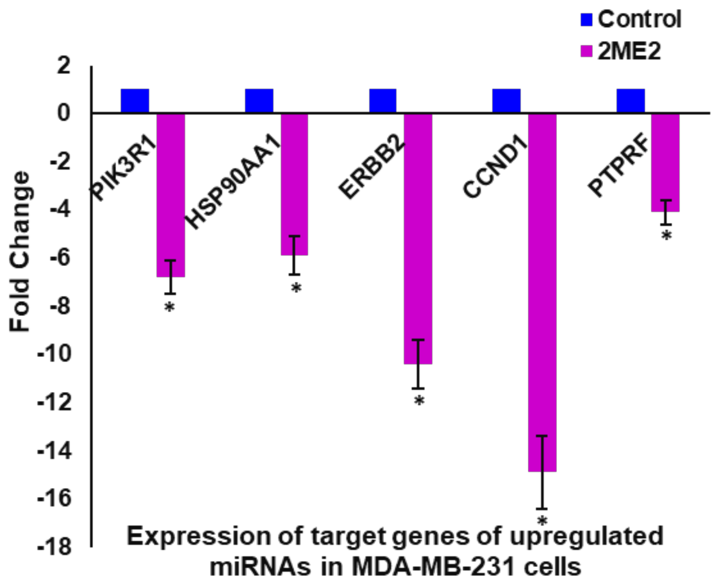

Supplementary Figure 7D

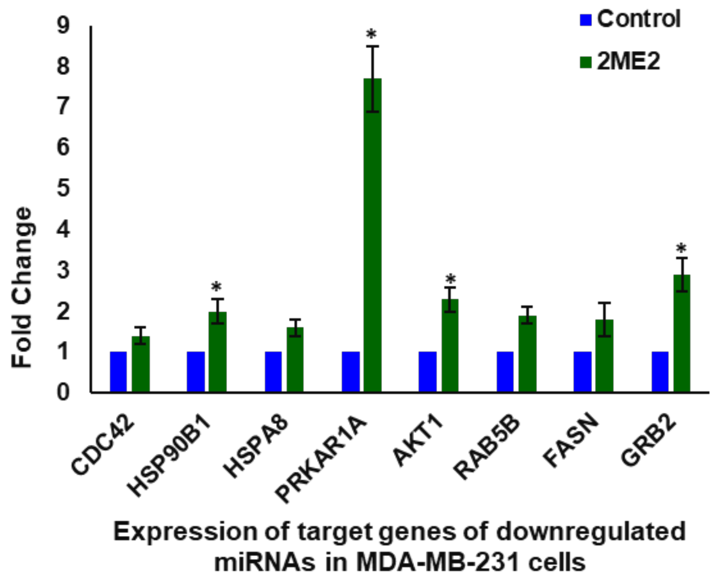

Supplementary Figure 7: A & B: RT-PCR analysis was performed to validate the expression of target genes of differentially regulated miRNAs in response to 2ME2 treatment in MDA-MB-468 cells. **C & D:** RT-PCR analysis was performed to validate the expression of target genes of differentially regulated miRNAs in response to 2ME2 treatment in MDA_MB-231 cells. The primers sequences of the target genes are mentioned in table 3.

Table 1: List of antibodies and dilutions

Antibody	Company	Dilution	Molecular weight (kDa)
pAKT S473	Cell Signaling 4060	1:1000	60
AKT1	Santa cruz 5298	1;500	62
pmTOR S2448	Cell Signaling 5536	1:500	289
mTOR S2448	Cell Signaling 4517	1:500	289
pP70S6K	Cell Signaling 9206	1:500	70-85
P70S6K	Cell Signaling 9202	1:500	70-85
E-cadherin	Cell Signaling 3195	1:500	135
N-cadherin	Cell Signaling 13116	1:500	140
Vimentin	Cell Signaling 5741	1:500	57
Slug	Cell Signaling 9585	1:500	30
Zeb1	Cell Signaling 3396	1:500	200
CDK4	BD Biosciences 610147	1:1000	33
CDK6	Cell Signaling 30483	1:500	36
Cyclin D1	Santa cruz 717	1:1000	37
P21	Abcam 7960	1:100	21
Cleaved PARP/PARP (Asp 214)	Cell Signaling 5625	1:1000	89-116
BAK	Cell Signaling 3814	1:1000	25
Bcl2	Santa cruz 783	1:500	26
Cleaved Caspase 3	Cell Signaling 9661	1:500	17-19
β-Actin	Sigma A1978	1:1000	42

Table 2: List of miRNA primers

**Top 5 up regulated miRNAs I**	**Forward**	**Reverse**
hsa-miR-4326	TGTTCCTCTGTCTCCCAG	GAACATGTCTGCGTATCTC
hsa-miR-646	5'-ACACTCCAGCTGGGAAGCAGCTGCCTC-3'	5'-
hsa-miR-600	CCACCTCTACGCATCATTCA	CCAAGCTCGTCTGGTTCTC
hsa-miR-2117	TGTTCTCTTTGCCAAGGAC	GAACATGTCTGCGTATCTC
hsa-miR-3200-5p	AATCTGAGAAGGCGCACAAG	GAACATGTCTGCGTATCTC
**Top 5 downregulated miRNA**	**Forward**	**Reverse**
hsa-miR-19a-3p	GCGTGTGCAAATCTATGCAA	AGTGCAGGGTCCGAGGTATT
hsa-miR-15a-5p	5′-TAGCAGCACATAATGGTTTGTG-3′	5′-GAACATGTCTGCGTATCTCAC-3′
hsa-miR-21-3p	GACCCAACACCAGTCGATG	TCCTCCTCTCCTTCCTTCTC
hsa-miR-19b-3p	GTGCAAATCCATGCAAAACTGA	GTGCAGGGTCCGAGGTGCT
hsa-miR-96-5p	ATGCTTTCTCAACTTGTTGG	TCACCG CTCTTGGCCGTCACA

Table 3: List of miRNA target genes primers

Genes	Forward	Reverse
PIK3R1	TGGACGGCGAAGTAAAGCATT	AGTGTGACATTGAGGGAGTCG
HSP90AA1	CAGAGGCGGACAAGAACGACAAG	GATCCTGTTGGCGTGCGTCTG
ERBB2	GGAAGTACACGATGCGGAGACT	ACCTTCCTCAGCTCCGTCTCTT
CCND1	TCTACACCGACAACTCCATCCG	TCTGGCATTTTGGAGAGGAAGTG
PTPRF	ATGTCATCGCCTACGACCACTC	GTGGCGATGTAGGCATTCTGCT
CDC42	TGACAGATTACGACCGCTGAGTT	GGAGTCTTTGGACAGTGGTGAG
HSP90B1	GGAGAGTCGTGAAGCAGTTGAG	CCACCAAAGCACACGGAGATTC
HSPA8	ACTCCAAGCTATGTCGCCTTT	TGGCATCAAAAACTGTGTTGGT
PRKAR1A	GCAGGCGAGCTATTAGTTTA	CATCCATCTCCTATCCCCTTT
AKT1	TGGACTACCTGCACTCGGAGAA	GTGCCGCAAAAGGTCTTCATGG
RAB5B	GGAGACTTCAGCCAAGACAGCT	ACACTGGCTCTTGTTCTGCTGG
FASN	TTCTACGGCTCCACGCTCTTCC	GAAGAGTCTTCGTCAGCCAGGA
GRB2	GAAATGCTTAGCAAACAGCGGCA	TCCATCTCGGAGCACCTTGAAG
GAPDH	GATGCTGGCGCTGAGTACG	GCTAAGCAGTTGGTGGTGC
